# Pedestrian detection algorithm integrating large kernel attention and YOLOV5 lightweight model

**DOI:** 10.1371/journal.pone.0294865

**Published:** 2023-11-29

**Authors:** Yuping Yin, Zheyu Zhang, Lin Wei, Chao Geng, Haoxiang Ran, Haodong Zhu

**Affiliations:** 1 Faculty of Electrical and Control Engineering, Liaoning Technical University, Huludao, Liaoning, China; 2 The Department of Basic Education, Liaoning Technical University, Huludao, Liaoning, China; 3 School of Software, Liaoning Technical University, Huludao, Liaoning, China; 4 School of Electronic and Information Engineering, Liaoning Technical University, Huludao, Liaoning, China; National University of Sciences and Technology NUST, PAKISTAN

## Abstract

In the context of intelligent driving, pedestrian detection faces challenges related to low accuracy in target recognition and positioning. To address this issue, a pedestrian detection algorithm is proposed that integrates a large kernel attention mechanism with the YOLOV5 lightweight model. The algorithm aims to enhance long-term attention and dependence during image processing by fusing the large kernel attention module with the C3 module. Furthermore, it addresses the lack of long-distance relationship information in channel and spatial feature extraction and representation by introducing the Coordinate Attention mechanism. This mechanism effectively extracts local information and focused location details, thereby improving detection accuracy. To improve the positioning accuracy of obscured targets, the alpha CIOU bounding box regression loss function is employed. It helps mitigate the impact of occlusions and enhances the algorithm’s ability to precisely localize pedestrians. To evaluate the effectiveness of trained model, experiments are conducted on the BDD100K pedestrian dataset as well as the Pascal VOC dataset. Experimental results demonstrate that the improved attention fusion YOLOV5 lightweight model achieves an average accuracy of 60.3%. Specifically, the detection accuracy improves by 1.1% compared to the original YOLOV5 algorithm, and the accuracy performance index reaches 73.0%. These findings strongly indicate the proposed algorithm in significantly enhancing the accuracy of pedestrian detection in road scenes.

## Introduction

Object detection technology [1] is a fundamental task in the field of computer vision, and the advancement of deep learning algorithms has greatly propelled the progress of intelligent driving technology [2]. Specifically, pedestrian object detection has emerged as a critical research area within the realm of autonomous driving, offering vast application prospects. Recent developments in the field of vision research have placed a greater emphasis on visual tasks such as pedestrian tracking [3], re-recognition [4], and UAV applications [5], leading to higher expectations for pedestrian target detection algorithms in terms of real-time performance, precision, and robustness. In conventional target detection algorithms, pedestrian detection primarily relied on manual feature extraction. These approaches often suffered from limited effectiveness in feature representation, leading to the adoption of more intricate methods for feature extraction and classification in traditional algorithms. Examples include the Viola-Jones detector [6], which employed complex feature representation techniques, and the gradient direction histogram (HOG) [7], which captured local shape information. However, these traditional detection methods often exhibited drawbacks such as a lack of targeted region selection strategies, resulting in prolonged processing times, window redundancy, and reduced accuracy.

Deep learning has emerged as the predominant technique in the field of object detection, leveraging the self-learning capabilities of deep convolutional neural networks to extract features from large-scale image data. These networks exhibit remarkable accuracy and robustness. Ongoing research efforts in deep learning have led to continuous improvements in the accuracy and performance of target detection algorithms. Notably, recent advancements have been made in processing image information through automatic feature learning using multi-layer neural networks. Two-stage algorithms, such as Faster R-CNN [8] and Mask R-CNN [9,10], have demonstrated exceptional detection accuracy. These methods employ selective search algorithms or regional proposal networks to extract region proposals, which are then utilized for target detection. Although these methods yield improved detection accuracy compared to traditional approaches, they suffer from complexity, high computational costs, and are unsuitable for real-time applications. Alternatively, single-stage target detection methods, exemplified by the Single Shot Detector (SSD) [11], offer faster detection speeds compared to two-stage methods. The network architecture of single-stage target detection algorithms has undergone continual refinement, with the introduction of notable algorithms such as YOLOV3 [12] (You Only Look Once) and YOLOV4 [13,14]. These algorithms utilize Darknet-53 as their base network and employ multi-level feature fusion techniques to enhance model accuracy. In the realm of real-time applications, several studies have made notable contributions to enhance the accuracy and efficiency of pedestrian detection. Chen et al. [15] addressed model flexibility and scalability challenges by improving the accuracy and speed of congested pedestrian detection through feature module enhancement and structural reparameterization. Meanwhile, in [16], the scholars introduced the Focal Loss into the RetinaNet [17] network. By appropriately balancing positive and negative sample weights, RetinaNet effectively mitigates the issue of category imbalance, thus improving detection accuracy. In order to focus more on the features in visible areas of pedestrians, in [18], the scholars devised a pedestrian detection network based on an attention mechanism. This approach enables the network to better handle the detection of occluded pedestrians and emphasize information from visible areas. Additionally, to tackle the challenge of minimal differences between different defects, in [19] the scholars proposed a fine coordinate attention module that encodes both average and significant information. This module facilitates capturing spatial dependencies and enabling long-range interactions. Recognizing the significance of a reduced number of significant features for model compression, in [20], the scholars introduced a normalization-based attention module to attenuate the importance of less significant features. They further applied a sparse weight penalty to enhance computational efficiency while maintaining performance. And the scholar like Cai et al. [21] introduced the pedestrian detection tasks in a cross-domain direction. They utilized the YOLOV5 detection model studying from an environment with abundant labels to one with sparse labels. The cross-conflict between target domain and source domain and the solution of image-level cross-domain alignment are studied in the background and foreground misalignment. And also there is the use of different shoot equipment to track and detecte pedestrians, like the use of Lidar to obtain 3D point cloud analysis,which is different from the general 2D space image matching algorithm. The scholars Meyer et.al, [22] point cloud analysis of pedestrians characteristics and automatic driving in the three-dimensional space, which is challenging for some real-time detection decision-making research methods.

The aforementioned pedestrian detection methods enrich the network structure through feature enhancement and the addition of attention modules, aiming to improve the accuracy of pedestrian detection tasks. However, accurate detection of pedestrian targets remains challenging due to the limited significance of target features and inaccuracies caused by environmental occlusions, leading to issues of missed detection and false detection. Consequently, to address these challenges inherent in pedestrian detection, this paper proposes an algorithm that integrates large kernel attention and the lightweight YOLOV5 model. The key scope of this approach are as follows:

1. To address the chanllenge of capuring long-range dependencies among images features and enhancing their semantic information, the YOLOV5 incorporating the Large Kernel Attention(LKA) module into the C3 module for feature fusion. Additionally,to reduce model complexity while preserving detection accuracy and speed, lightweight GhostConv module is employed for compression.
2. To enhance the spatial perception performance and improve the accuracy of pedestrian detection task, a novel framework called Coordinate Attention with Normalization Convolution Block Attention Module (CA_NCBAM) is proposed. It facilitates an improved understanding of the relative relationship between different positions in the image. It utilizes multiple descriptors and spatial attention to effectively supplement channel attention, while accounting for key location information and the correlation between channels.The method enables the network to focus more accurately on target objects and extract local information and attention location information, thereby enhancing the detection accuracy of the algorithm. By considering both the key location information and the correlation between channels, the proposed approach is able to capture fine-grained details and better distinguish between objects in complex scenes.
3. To enhance the accuracy of the bounding box regression in object detection, the alpha CIOU denoted as loss function. It further selects suitable values for the parameter alpha and applies a gradient adaptive reweighting method in the existing CIOU loss function. This approach aims to accelerate inference time while maintaining the accuracy.

### Basic principles of the YOLOV5 algorithm

**Fig 1:**
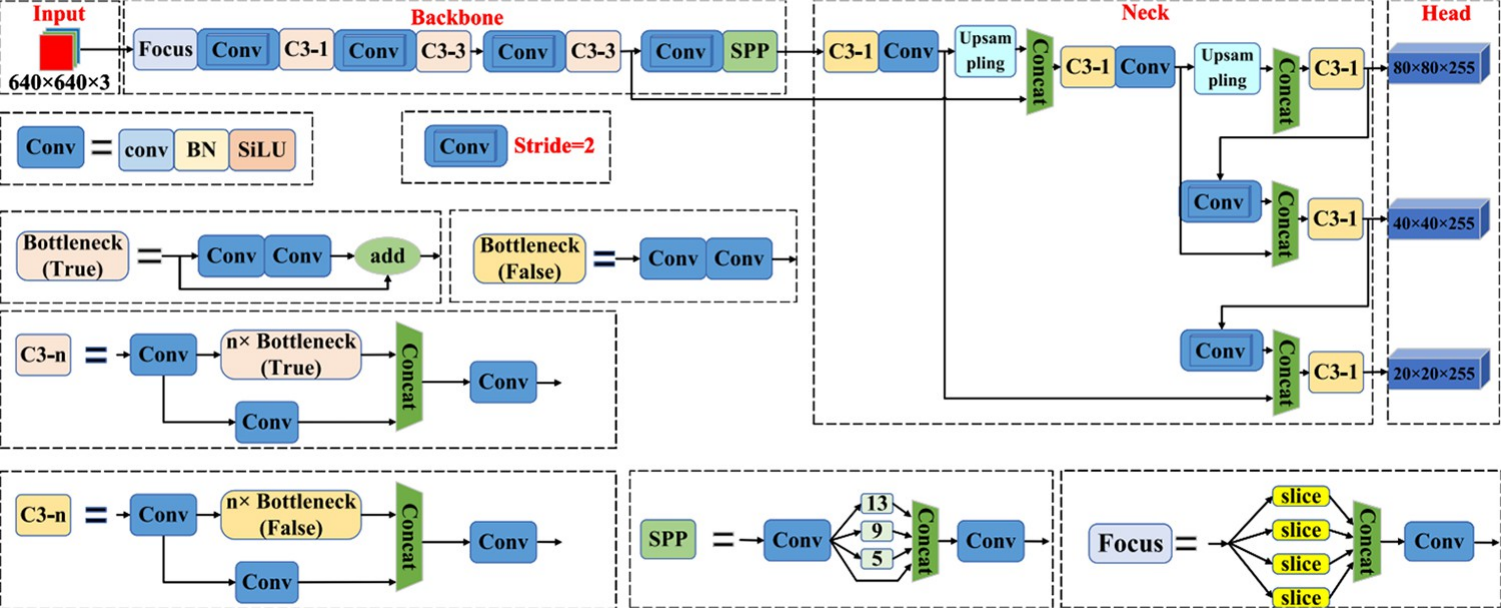
The architecture of the YOLOV5 mothod.

The YOLOV5 algorithm encompasses four models based on the network depth and feature map width. Among these models, YOLOV5s exhibits the fastest processing speed, while YOLOV5x achieves the highest detection accuracy. Structurally, all four models share a common network architecture comprising an input terminal, backbone, neck network, and detection head. The input stage involves operations such as mosaic data augmentation, image size processing, and adaptive anchor box computation. While the image enters the network through the input terminal, it undergoes downsampling and other operations in the backbone network before being propagated to the neck network. Within the neck network, both the feature pyramid network (FPN) and path aggregation network (PAN) facilitate feature fusion and extraction of image features at varying scales. FPN establishes multi-level connections to transmit semantic information, enabling the network to process objects across multiple scales. On the other hand, PAN focuses on enhancing the localization and expressive capabilities of low-level features, thereby improving the network’s accuracy and robustness. The cooperative interaction between these two feature pyramid models strengthens the fusion effect within the neck network. By utilizing these structural components and mechanisms, the YOLOV5 algorithm aims to optimize the fusion of features and extract relevant information at different scales. To cater to the detection of target objects of varying sizes, the YOLOV5 algorithm employs three detection layers of different sizes in its detection head. These layers output essential information such as category probabilities, object scores, and bounding box positions, which collectively enable the generation and identification of predictive bounding boxes and their corresponding categories. This process facilitates the accurate identification of target objects within the image. The architecture of the YOLOV5s network model is illustrated in Fig 1.

**Fig 2:**
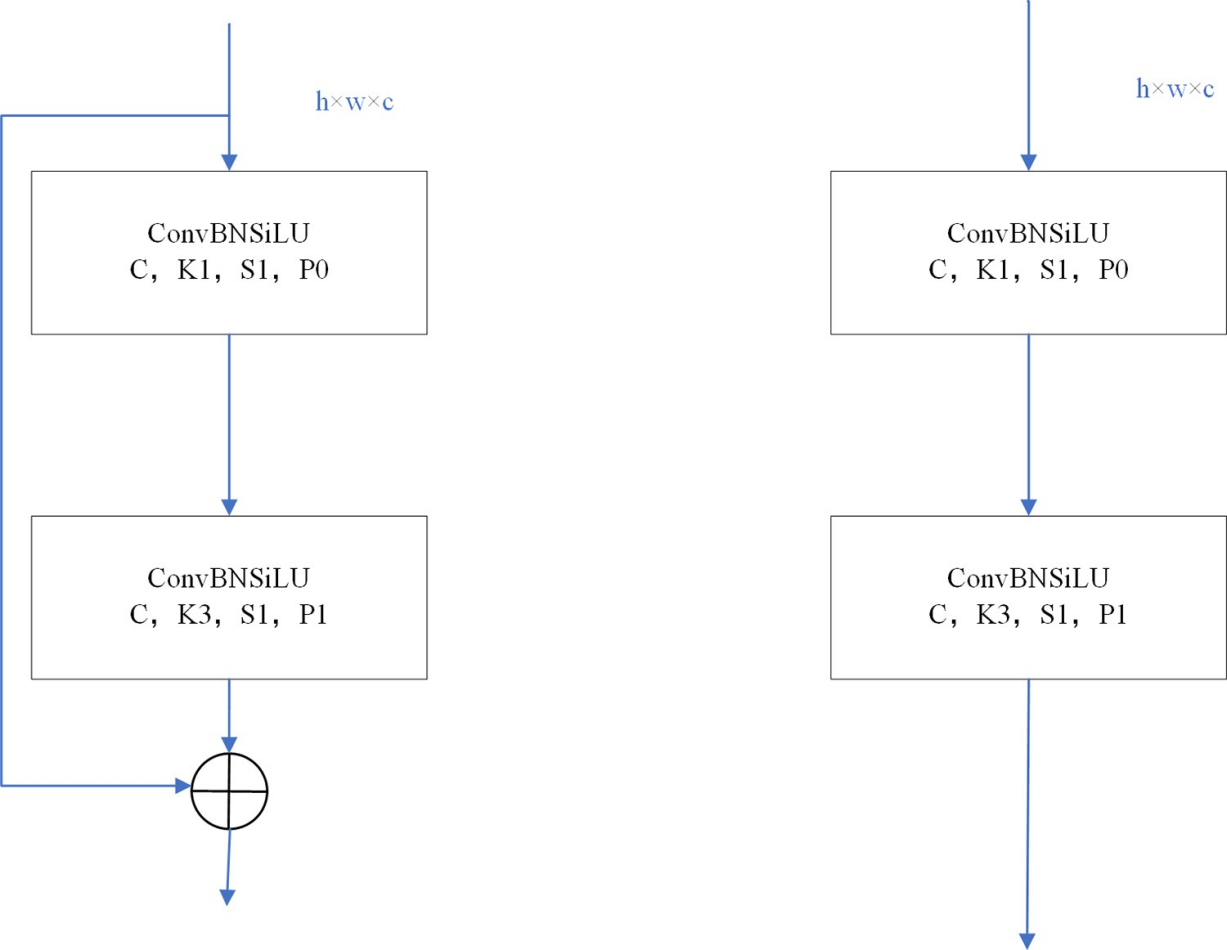
Bottleneck network in C3 module.

The backbone network of YOLOV5s comprises several key modules, including the focus module, Conv block, C3 module, and space pyramid pooling (SPP) module. The focus module serves a dual purpose of increasing channel size and speed while also implementing downsampling operations. On the other hand, the Conv block represents the fundamental unit of convolution within the YOLOV5s architecture. It consecutively applies convolution, normalization, and activation function rules to the input. The C3 module is based on a bottleneck design, as depicted in Fig 2. Additionally, the SPP module enhances receptive field of the YOLOV5s network by performing maximum pooling using different kernel sizes and merging the resulting features. This approach enlarges the sensitivity field of the network model by incorporating multiple pool kernel sizes. Moreover, the SPP module leverages translation invariance to alleviate optimization challenges and reduce the number of parameters. By integrating these components and modules, the YOLOV5s model effectively tackles the task of object detection, providing the capability to identify target objects across a broad range of sizes.

### Pedestrian detection algorithm integrating large kernel attention and YOLOV5 lightweight model

**Fig 3:**
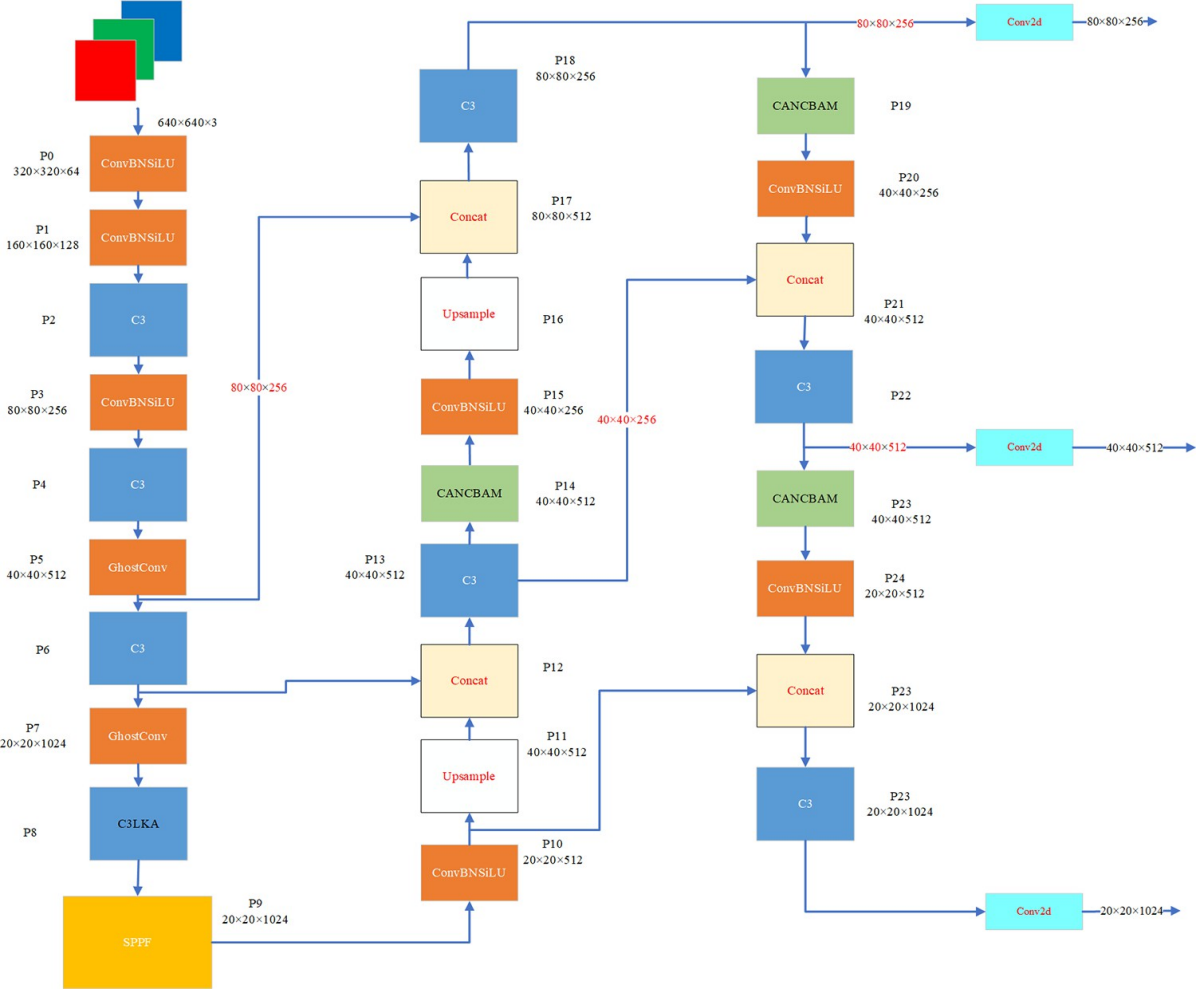
The architecture of the improved YOLOV5 mothod.

Considering the challenges posed by traditional detection algorithms, such as inadequate pedestrian recognition, positioning, and detection rates in unmanned driving scenarios, as well as missing due to occlusions from the surrounding environment, this paper introduces a pedestrian detection algorithm integrating large kernel attention and YOLOV5 lightweight model. The proposed model structure is illustrated in Fig 3. In this approach, YOLOV5s serves as the baseline model, ensuring high detection accuracy while simultaneously reducing the number of parameters and model size. To address the issue of insufficient local information in target detection, a large kernel attention fusion C3 module has been employed. This module is designed to capture contextual information and establish long-distance relationship dependencies, which are critical factors for accurate target detection. Additionally, a CA_NCBAM hybrid attention module has been integrated into the detection task to facilitate key information selection, thereby improving the efficiency and accuracy of image information processing. Then, to ensure that the network better acquires depth information from the image for pedestrian detection location analysis, loss function reweighting optimization has been utilized.

### C3 module embedded with large kernel attention mechanism

Within convolutional neural networks, shallow features are adept at extracting intricate spatial details, such as textures and edges, thereby proving beneficial for object localization tasks. Conversely, shallow features tend to exhibit limited performance in object classification tasks due to their lack of comprehensive global semantic information. On the other hand, high level features within the convolutional neural networks possess richer semantic information, enabling them to effectively differentiate objects belonging to distinct categories. However, the extraction of deep features often results in the loss of significant spatial details. To address this challenge, Guo et al. [23] introduced the visual attention network (VAN), which emphasizes the adaptability of local structural information and channels in visual tasks. Remarkably, VAN achieves comparable performance to vision transformer in image processing tasks, thus showcasing its advanced capabilities. In order to improve the detail sensitive tasks and accuracy of the model for small target detection, and select the key information in the detection task, this paper combines the large kernel attention and C3 module to form a new C3LKA module, and uses the rotational invariance of the local receptive field during convolution operation and the long-distance information dependence of the self-attention mechanism to improve the expression ability of the convolutional neural network. It can better capture global semantic information and local context information, and consider more linear complexity and dynamic processes.

**Fig 4:**
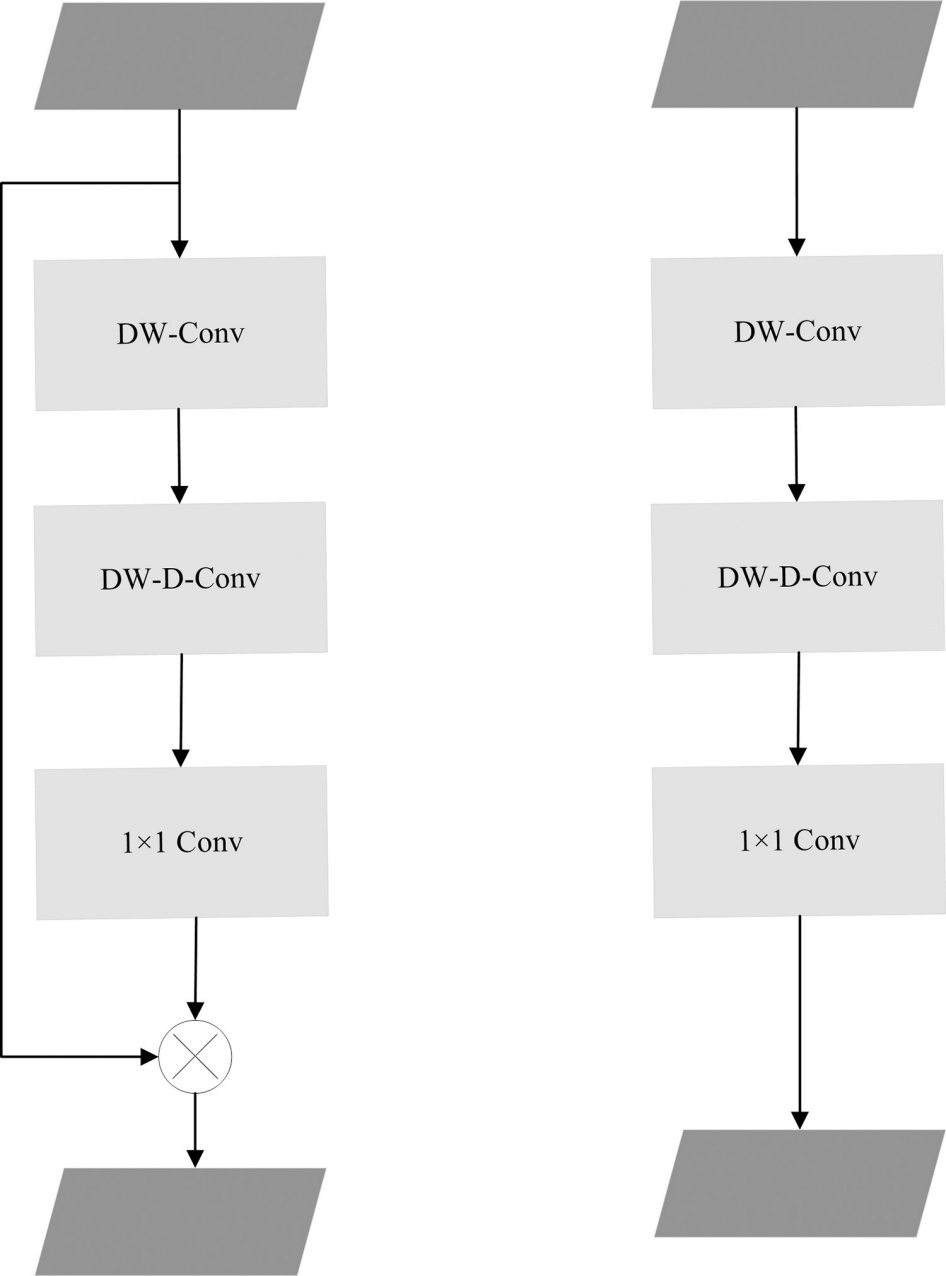
Structure diagram of two LKA modules.

The YOLOV5 model incorporates the large kernel attention (LKA) mechanism, which ensures adaptability in both the channel and spatial dimensions. The LKA can be comprehensively understood through the following three components, as illustrated in Fig 4, a spatial local convolution for spatial depthwise processing, a spatial long-range convolution for capturing long-distance spatial relationships, and a 1×1 channel convolution. Specifically, the 21×21 large kernel convolution is decomposed into a 7×7 deep dilation convolution with a dilation coefficient of 3, and a 5×5 deep convolution followed by a 1×1 convolutional operation. The calculation procedure of the LKA module is detailed in Eqs (1) and (2):

$$Attention=Conv_{1\times 1}(DW•D•Conv(DW•Conv(F)))$$ (1)

Output=Attention⊗F (2)

within the input feature map *F*∈*R*^*C*×*H*×*W*^, attention expression is *Attention*∈*R*^*C*×*H*×*W*^, ⊗ achieved by multiplying each element, *DW*•*Conv*is through the utilization of contextual information within the image to emphasize local structural features. Furthermore, *DW*•*D*•*Conv* facilitates a focus on long-term information and establishes long-distance dependencies. Notably, LKA differentiates itself from other attention mechanisms by amalgamating the strengths of convolution and attention, obviating the requirement for activation functions such as Sigmoid or Softmax. With less calculation, LKA attains long-term attention and associated parameters while generating the attention maps based on the significance of pertinent estimation points. Consequently, it can adaptively adjust the output based on input characteristics, thus ensuring adaptability in both spatial and channel dimensions.

In this paper, the LKA module is selected to replace the original bottleneck module, resulting in the creation of a novel C3LKA module for convolution. This modification enables the model to prioritize the local context information within images, expanding the receptive field, and accommodating linear complexity and dynamic processes. Additionally, the Ghost module is employed to partition the original convolution layer into two segments, employing a smaller number of convolutional filters to generate internal feature maps. By ensuring model accuracy, the approach effectively reduces the number of parameters and computational burden. The primary objective of this strategy is to mitigate the computational cost associated with convolution operations, thereby facilitating more efficient model training and inference.

**Fig 5:**
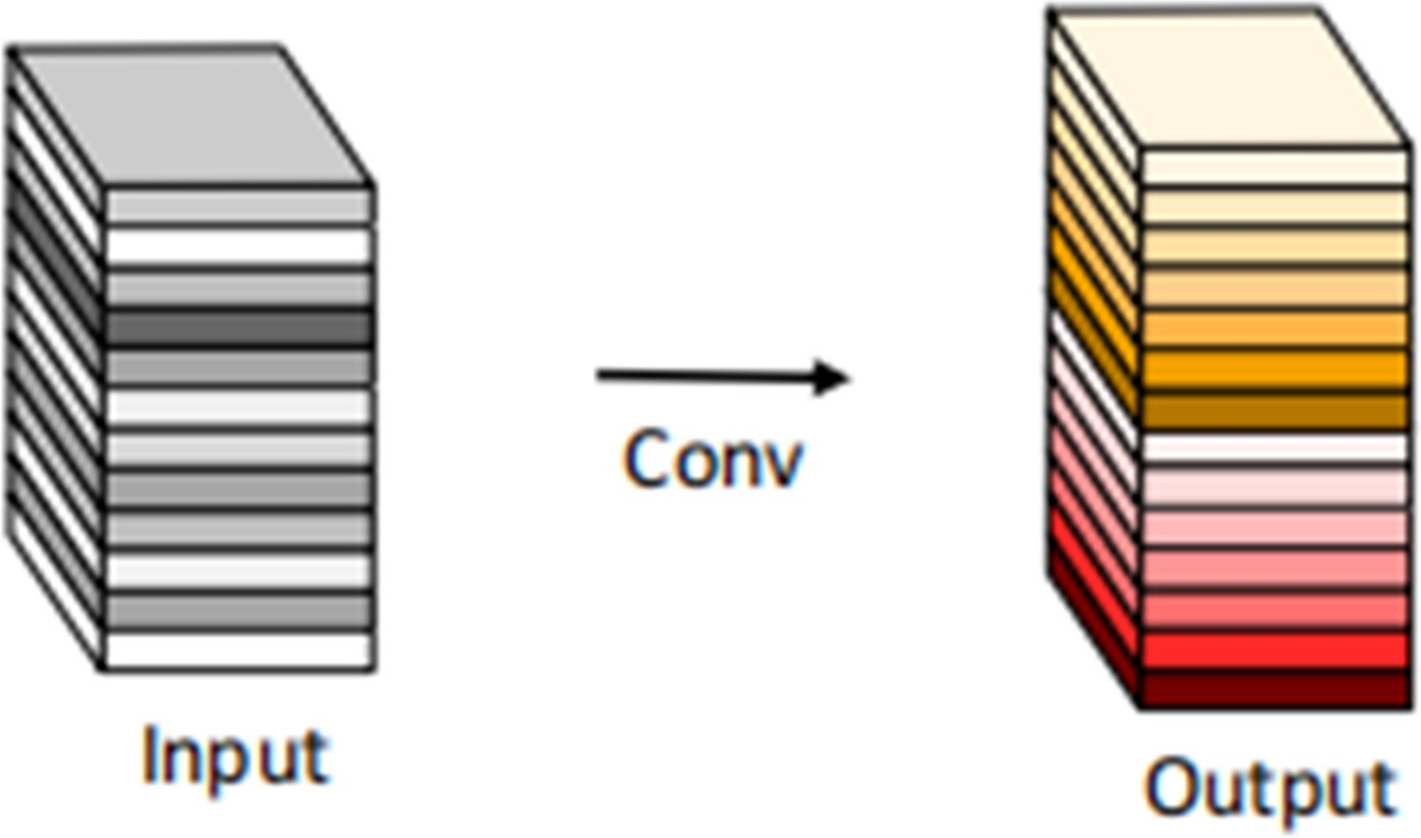
The convolutional layer.

**Fig 6:**
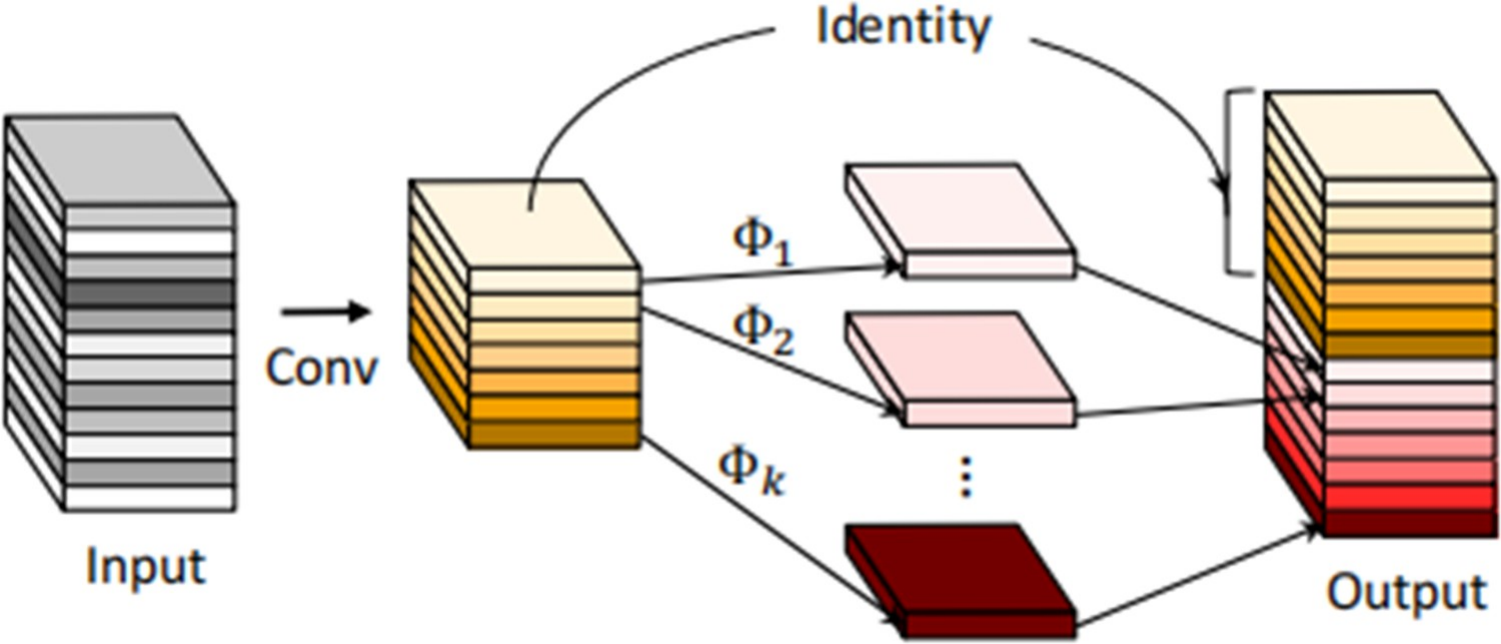
The Ghost module.

Specifically, the Ghost convolution [24] is implemented by partitioning the input channel into two segments: a smaller portion and a larger portion. The smaller portion undergoes the standard convolution operation, while the larger portion undergoes a reduced convolution operation using a smaller kernel size as 5×5. By employing this approach, calculations can be performed for each output channel, thus reducing the computational complexity and thus further reducing the model size and computational requirements. Group convolution is utilized to divide the input channel into multiple distinct groups, enabling independent convolution computations within each group. This division effectively reduces the number of parameters and computational burden of the model while enhancing the computational efficiency of the convolutional layer. The schematic diagrams depicting the Ghost convolution are presented in Figs 5 and 6.

The Ghost convolution procedure involves multiple steps. Firstly, the input feature map *X*∈*R*^*c*×*h*×*w*^ undergoes conventional convolutional feature extraction, resulting in a smaller output tensor. Subsequently, the Ghost convolution operation is performed, generating a larger output segment. Finally, the two output parts, obtained from the Ghost convolution and the original convolution, are concatenated along the channel dimension, yielding the final output feature map *Y*∈*R*^*c*×*h*×*w*^. During the convolution process, the amount of floating-point arithmetic involved is represented as *c*×*h*’×*w*’×*c*’×*k*×*k*. Comparatively, when the same number of features is output, the Ghost convolution operation reduces redundancy in comparison to regular convolution. By effectively reducing computational costs, the Ghost module accelerates linear reasoning operations. The calculation rate between them can be derived as Formula (3).

$$\begin{array}{l} r_{s}=\frac{c\times h\times w\times c^{'}\times k\times k}{\frac{c}{s}\times h\times w\times c^{'}\times k\times k+(s-1)\times \frac{c}{c^{'}}\times h\times w\times d\times d}\\ \approx \frac{s\times c^{'}}{s+c-1}\approx s \end{array}$$ (3)

with *k*×*k* representing the size of the convolution filter, and the convolution kernel size for linear operation is denoted as *d*×*d*. Additionally, *s* denotes a simple conversion operation and *s*≪*c*’. From Eq (3), it is evident that the convolution operation performed by the Ghost module requires 1/*s* times fewer calculations compared to the standard convolution calculation. This observation highlights the ability of the Ghost module to effectively reduce computational costs and compress the model size. By incorporating the Ghost module, the network model becomes more lightweight, enabling seamless integration and deployment on mobile devices. The Ghost module is designed and optimized to accommodate deep separable convolution and scale it for specific tasks. These optimizations contribute to a lighter model, making it well-suited for mobile deployment scenarios.

### Coordinate fusion CBAM attention module

With the objective of addressing the issue of the network’s attention towards visible areas of pedestrians on the road, in [25], the scholars proposed the utilization of the channel attention mechanism SENet(Squeeze-and-Excitation Networks), to enhance classification performance. The principle involves augmenting the weight relationships among feature channels pertaining to the visible regions in the image. By doing so, the model gains an improved ability to capture crucial information within the image, better comprehending the relationship between distinct channels and positions. Consequently, the model’s accuracy have been enhanced. To accurately focus on the target object, in [26], the scholars introduced a Convolutional Block Attention Module (CBAM) that is both straightforward and effective. This module effectively subdivides intermediate features by either emphasizing or inhibiting image information, enabling the network to correctly concentrate on the target object. In addition to channel dimension focus, a spatial attention module is integrated to emphasize the spatial structural characteristics across diverse locations within the image. This aids in enhancing the model’s performance in recognition tasks and enhances its robustness. Furthermore, the scholars proposed a Global Attention Mechanism module in [27], which aims to improve the performance of neural networks by minimizing information reduction and amplifying global information interaction. And it attempts to simplify parameters to achieve cross-dimensional attention, thus enhancing the network’s overall capabilities.

**Fig 7:**
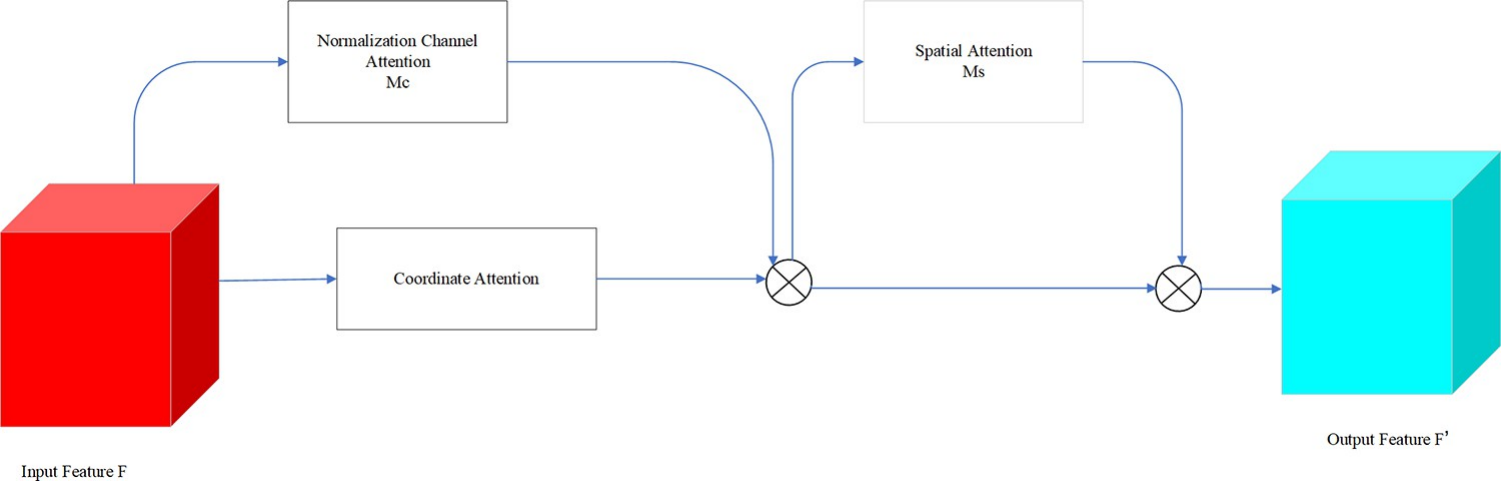
The framework of CA_NCBAM.

To address the issue of incomplete pedestrian features and the detection of small targets in pedestrian detection, this paper leverages the advantageous features of the CBAM module and integrates them into the proposed methodology. By redesigning the channel attention submodule and incorporating the coordinate attention module, the model introduces a lightweight and efficient attention mechanism known as CA_NCBAM. This novel attention mechanism aids in enhancing the model’s ability to learn intricate relationships among various positions within the image, thereby improving its overall generalization capability. The structure of the attention fusion module is observed in Fig 7.

To enhance the performance of the network across diverse tasks, a scaling factor is introduced to adjust the weights of different channels within the channel attention module. It helps the network to effectively allocate resources and prioritize the most relevant channels. A sparse regularization term is incorporated into the attention module. This regularization term promotes the activation of a smaller subset of attention heads while suppressing others, thereby reducing computational requirements while maintaining comparable performance levels. The batch normalized scaling factor is employed to quantify the variations between different channels, serving as a measure of their importance. Channels exhibiting significant changes indicate a higher richness and importance of the information they contain. Conversely, channels with minimal changes suggest a more limited and less crucial information content. The calculation of the scale-factor within the batch normalization process is depicted by Eq 4.

$$B_{out}=BN(B_{in})=\gamma \frac{B_{in}-\mu_{B}}{\sqrt{\sigma_{B}^{2}+\varepsilon }}+\beta$$ (4)

With *μ*_*B*_ and *σ*_*B*_ representing the mean and standard deviation of a given mini-batch B, respectively. The trainable scale and displacement transformation parameters are represented by *γ* and *β*. In the context of pixel normalization, scale factors are employed to indicate the significance of individual pixels. The expression of the channel attention submodule is formulated as shown in Eq 5.

$$M_{c}=sigmoid(W_{\gamma }(BN(F_{1})))$$ (5)

**Fig 8:**
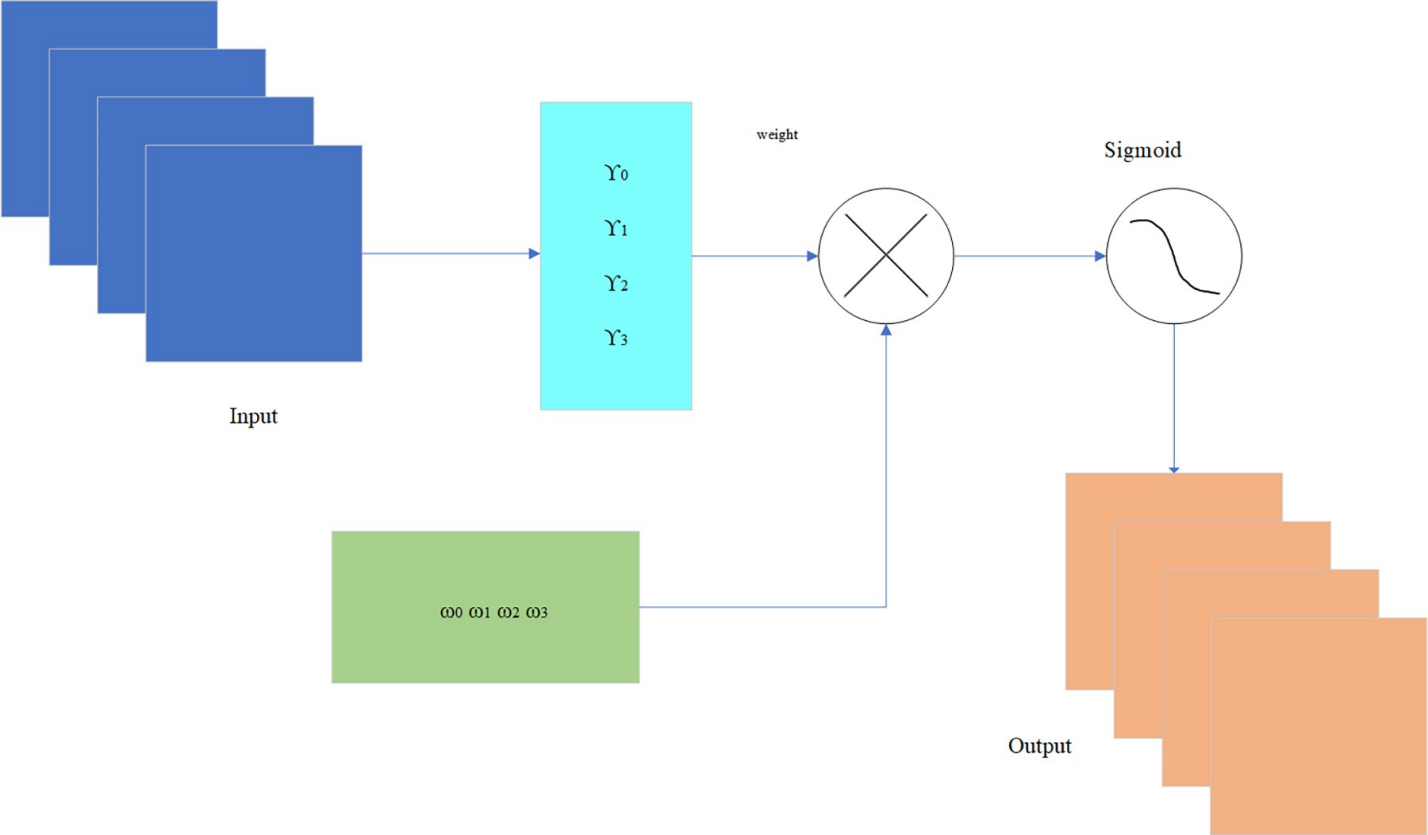
Normalization channel attention module.

Normalization channel attention involves compressing the feature map along the spatial dimension, resulting in a one-dimensional vector representation. During this compression, the dimension of the feature map is transformed into *C*×1×1 by employing parallel average pooling and maximum pooling operations, which are served to aggregate the spatial information within the feature map and transmit it to a shared network, thereby compressing the spatial dimension of the input feature map. By performing element-wise summation and merging, channel attention maps are generated through the application of different weight effects via the scaling factor. The resulting channel attention maps exhibit distinct features across different channels, as depicted in Fig 8.

Normalized channel attention can be expressed as Formula (6):

$$M_{c}(F)=\sigma (W_{\gamma }(BN(MLP(AvgPool(F))+MLP(MaxPool(F)))))$$ (6)

with *M*_*c*_ being output feature and *γ* representing the scale factor of each channel. The weight expression is *W*_*γ*_ = *γ*_*i*_/∑_*j* = 0_*γ*_*j*_.

The spatial attention mechanism is a technique utilized for channel compression. It applies average pooling and maximum pooling operations along the channel dimensions, resulting in 1×*H*×*W* feature maps. In contrast to channel attention, spatial attention focuses on the significance of location information within the image. By assigning weights to the spatial features, the spatial attention mechanism is able to emphasize the more crucial regions of the image. Consequently, it enables the selection of pertinent local information from the input image, thereby serving as a valuable complement to the channel attention module. The mathematical expression for the spatial attention mechanism is presented in Eq (7).

$$M_{s}(F)=\sigma (f^{7\times 7}([Avgpool(F);MaxPool(F)]))$$ (7)

with *σ* the activation function, *f*^7×7^ the convolution operation with a size of 7×7. and the characteristic values *AvgPool*(*F*) and *MaxPool*(*F*) are after average pooling and maximum pooling. In the context of pedestrian target detection, enhancing the efficiency and accuracy of target detection requires reinforcing relevant information while suppressing irrelevant feature information.

In order to better understand the position and shape of objects in the image, it is achieved by encoding the channel relationships, which enables the extraction of precise position information and long-term dependencies in object understanding. In this paper, the integration of Coordinate Attention with the CBAM module is introduced to incorporate coordinate information. This integration allows them to accurately position image coordinate information and extract more effective target information features, enhancing the model’s recognition capabilities while focusing on both spatial and channel attention.

The channel attention is decomposed into two distinct one-dimensional feature encoding processes that aggregate features along two spatial directions. One process captures long-range dependencies along the horizontal spatial direction, while the other process preserves precise location information along the vertical spatial direction. Consequently, the resulting feature maps are encoded as a pair of attention weight maps, a direction-aware attention map *f*^*h*^∈*R*^*C*/*r*×*H*^ and a location-sensitive attention map *f*^*w*^∈*R*^*C*/*r*×*W*^. These attention maps are applied complementarily to the input feature maps *f*∈*R*^*C*/*r*×(*H*+*W*)^ in order to enhance the representation of the target object. Finally, it is performed on these attention maps using a sigmoid activation function for feature transformation, ensuring the consistency of the output feature graph.

### Loss function

The loss function in YOLOV5 mainly includes three kinds of losses,namely category loss (to calculate the classification loss of positive samples), confidence loss (to calculate binary cross entropy) and positioning loss, which are used to calculate the distance between the network prediction information and the expected information. When the prediction information is closer to the expected information, the loss function value is smaller. The total loss function is as shown in Eq (8):

$$Loss=\lambda_{1}L_{cls}+\lambda_{2}L_{obj}+\lambda_{3}L_{loc}$$ (8)

The loss function of YOLOV5 is CIOU (complete IOU loss), which takes into account the aspect ratio of the bounding box into the loss function based on DIOU (distance IOU loss) and adds an influence factor to the original base penalty term to further improve the detection regression accuracy. the CIOU calculation formula is as follows Eqs (9)–(11):

$$v=\frac{4}{\pi^{2}}{\left(\arctan\frac{w^{gt}}{h^{gt}}-\arctan\frac{w}{h}\right)}^{2}$$ (9)

$$\alpha =\frac{v}{1-IOU+v}$$ (10)

$$CIOU=IOU-\frac{\rho^{2}(b,b^{gt})}{c^{2}}+\alpha v$$ (11)

with *ρ*^2^(*b*,*b*^*gt*^) being the square of Euclidean distance between the prediction box and the real box, *c* the diagonal line of the smallest closed rectangle formed by the prediction box and the target box, and IOU is the intersection ratio relation between the two boxes. In this paper, alpha CIOU is used as the loss function, which can better unify the existing loss exponentiation of IOU, and better realize the precision regression of different boundary frames by adjusting the appropriate alpha () value. In order to retain the scale information of boundary frames, the method of relative order retention is used as a priori to obtain the optimal IOU function. In order to optimize the effect of loss function, the proportional loss gradient reweighting method was adopted to better optimize the position and size of the detection boundary frame and reduce the missed detection rate of the model. The better result is achieved with *α* = 3. The loss function of Alpha CIOU is calculated as Eq (12):

$$L_{\alpha -CIOU}=1-IOU^{\alpha }+{\left(\frac{\rho^{2}(b,b^{gt})}{c^{2}}\right)}^{\alpha }+{\left(\beta v\right)}^{\alpha }$$ (12)

where *α* and *v* denote the penalty term to CIOU. The alpha CIOU provides high performance detection expressions and adaptive weighting of bounding box gradients in high precision regression calculations, maintaining the model size while accelerating convergence, resulting in improved pedestrian detection accuracy and better regression results.

## Experimental results and analysis

### Data set and experimental setup

The training process for the experiments in this paper is running under the NVIDIA GeForce GTX 3080 Ti, the CPU used to training is Intel(R) Core(TM) i7-11700K, with 32.0GB of RAM and 12GB of video memory. The platform of this experiment is windows 10, CUDA 11.7 and cudnn 7.6.5. The algorithm is based on Python 3.9.10 and Pytorch 1.10.0. The image size for network training is set to 640×640, and the batch_size is set to 16, and data enhancement as Mosaic is used to increase the diversity of training samples and improve the generalization ability of the network. The momentum parameter is set to 0.937, with a weight decay factor of 0.0005, and 200 epochs are set for training cycles. To evaluate the improved model algorithm, Our experiment used two datasets for detection comparison, a publicly available BDD100K pedestrian dataset for autonomous driving and the widely used evaluation metric dataset Pascal VOC in this paper.

### Evaluation indicators

In this paper, precision and recall are used as evaluation indexes of model detection speed for quantitative evaluation of detection results, so as to more comprehensively verify the performance of the proposed improved YOLOV5s. In the experiment, precision, recall rate, mAP@0.5, parameter number and FLOPs are used as measurement indexes. Accuracy rate is the ratio between the number of correctly predicted positive samples and the number of predicted positive samples; recall rate is the proportion of all predicted correct targets, and the formulas are as follows in Eqs (13) and (14):

$$P=\frac{TP}{TP+FP}$$ (13)

$$R=\frac{TP}{TP+FN}$$ (14)

where TP refers the number of positive examples correctly classified, FP denotes the number of negative samples actually judged to be positive, FN indicates the number of negative examples correctly classified and wrong, TN is the number of negative examples correctly classified. The calculation of mAP@0.5 and AP is defined as follows in Eqs (15) and (16):

$$mAP=\frac{1}{N}\sum_{i=1}^{N}AP_{i}$$ (15)

$$AP=\int_{0}^{1}P(R)dR$$ (16)

where mAP@0.5 is an evaluation measure that computes the average AP for all categories when the IOU threshold is set to 0.5. FPS as a metric description of the image refresh frequency is an important measurement index for us in processing the video and image source file reasoning. And the average detection processing time includes preprocessing time, network inference time and non-maximum suppression (NMS) processing time, and the formulas are as follows in Eqs (17) and (18):

$$FPS=1000/T_{total}$$ (17)

$$T_{total}=t_{Pre}+t_{Inf}+t_{NMS}$$ (18)

and the model size refers to the size of the trained model saved after training.

### Experimental results on BDD100K dataset

#### BDD100K dataset

**Fig 9:**
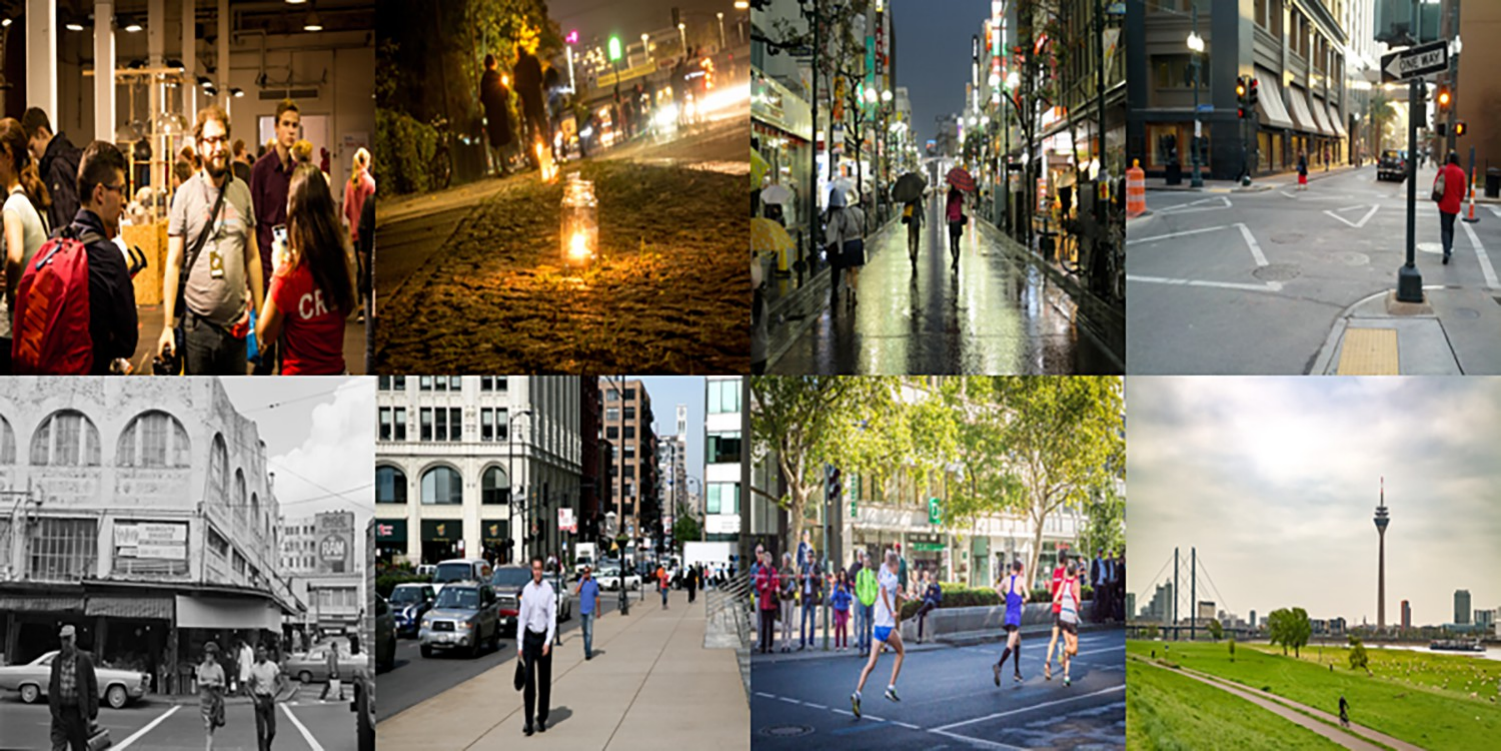
The Pedestrians images.

In order to evaluate the performance of the improved YOLOV5s in pedestrian detection, BDD100K data set is used in this paper for effect detection. The BDD100K dataset is published by the AI Lab in Berkeley, California, and is one of the largest and most diverse real-world autonomous driving data sets for street acquisition to date. The data set is shot in a variety of weather scenes and at different times, such as sunny days and clouds, residential areas and urban roads, day and night and other different scenes. The diversity of such data is extremely important for the robustness of the detection perception algorithm. There are 10 predefined classes in this data set, including Bus, Light, person, Car and other labels. This paper extracts 25320 person categories from the data set for experimental detection. Randomly extract the extracted data set and divide at ratio of 8:2. Before the experiment, the data set is divided into 4420 pictures for training set and 1127 pictures for verification set. The pedestrian images are shown in Fig 9.

#### Ablation experiment with an improved model

**Fig 10:**
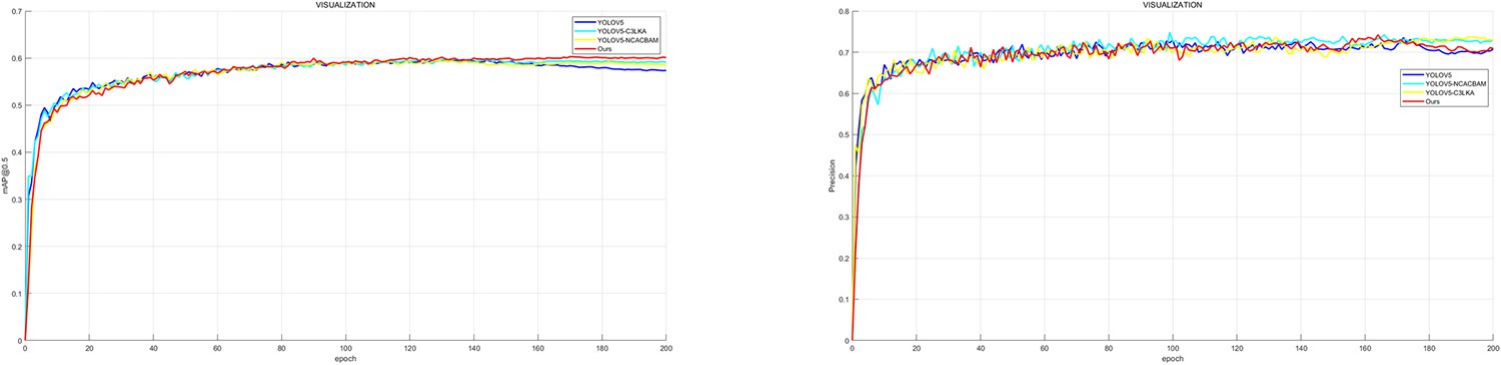
The experimental results of each model on BDD100K dataset. (a) The precision change curve (b) The curve of change in mAP@0.5.

In this section, a total of five experiments are trained, namely, YOLOV5, YOLOV5+C3_LKA, YOLOV5+CA_NCBAM, YOLOV5+alpha_CIOU, and the proposed model in this paper by ablation experiments to compare the performance changes caused by changes in network structure. The training process of the performance ablation experiments under different modules compared on the BDD100K pedestrian data set in Fig 10, and the experimental results are shown in Table 1.

**Table 1:** The results of BDD100K under different models.

Model	mAP@0.5 (%)	Precision (%)	Params (M)	Flop (G)	FPS
**YOLOV5**	59.3	71.6	7.01	15.8	153.84
**YOLOV5+C3_LKA**	59.7	72.5	11.27	19.2	89.29
**YOLOV5+CA_NCBAM**	59.5	73.1	7.05	15.9	101.01
**YOLOV5+alpha CIOU**	59.5	69.9	7.01	15.8	153.80
**ours**	**60.4**	73.0	10.72	18.8	80.65

**Fig 11:**
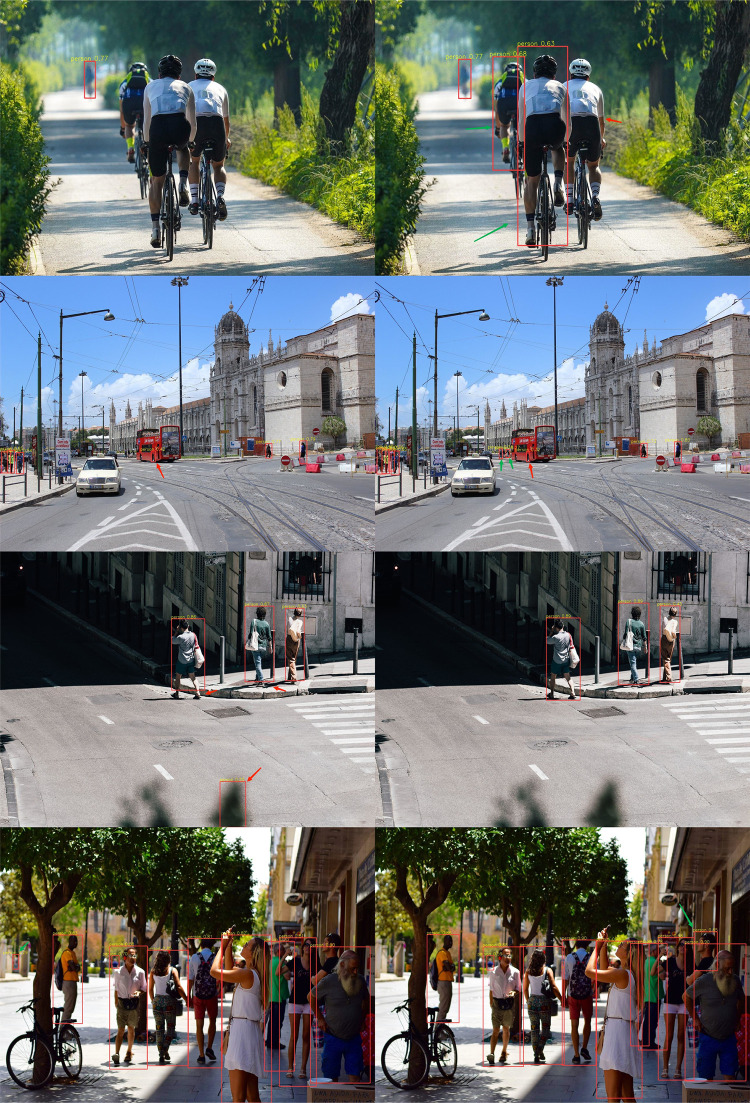
Experimental results of different scene models. (a) Result of YOLOV5 (b) Algorithm testing results in this paper.

As can be seen from Table 1, the accuracy of the improved model is increased by 1.1%, indicating that the improved module can effectively improve the accuracy of target detection and reduce the case of missing detection. The performance comparison between the proposed method and YOLOV5s is shown in Fig 11. The image on the left is the detection result of YOLOV5s, and the image on the right is the detection result of the proposed method.

In terms of everyday scenes and complete target pedestrian detection, the proposed algorithm deviates less from YOLOV5s detected target results for obscured and fuzzier small target pedestrians, as shown in Fig 11, where the method detects obscured pedestrians that are not detected by YOLOV5. The green arrows are targets not detected in YOLOV5 but detected by the improved method, and the red arrows pointed targets are those that YOLOV5 mistakenly detects targets and poor pedestrian targets. The results show that the improved algorithm outperforms YOLOV5s pedestrian target detection.

The ablation experimental evaluation results of the training model on the BDD100K [28] pedestrian data set are shown in Table 1. YOLOV5s_C3_LKA refers to the C3_LKA module introduced into the backbone architecture of YOLOV5s. In addition, CA_NCBAM module is used in the neck network in this paper. To highlight information helpful for pedestrian detection and suppress other useless information. As can be seen from Fig 11, the performance comparison of this method in simple scenes is basically the same as that of the benchmark model, but it has better detection performance compared with the benchmark model in occlusion and remote small target objects. The CIOU loss used in YOLOV5s has some shortcomings, such as inaccurate network regression and insensitivity to small objects due to the fact that it only contains the region information of the boundary box, so alpha CIOU loss is used to replace the original loss function, which considers that the property of alpha has better flexibility to the IOU adaptability of the target. By improving the regression accuracy and sensitivity of different horizontal boundary frames to small objects, the detection performance of the improved model is better than that of YOLOV5s.

The proposed algorithm was compared with some other pedestrian detection algorithms in the BDD100K pedestrian data set for horizontal comparison experiment. The mAP0.5, average recall rate and parameter model are used as performance evaluation indexes. In Table 2 the performance of different model d-etection methods. Combined, it can be seen that compared with Faster RCNN and YOLOV7, the improved algorithm increases 10.36% and 1.2% respectively, and the difference is not obvious compared with the average recall rate of other models with Table 2.

**Table 2:** Results of different models on BDD100K dataset.

Model	mAP@0.5	AR	Params(M)	FPS
**Faster R-CNN**	50.04%	53.10%	3.01	/
**YOLOV7**	59.20%	53.60%	36.48	76.3
**YOLOV3-SPP**	61.60%	55.9%	62.55	49.5
**YOLOV5**	59.30%	52.60%	7.01	153.8
**YOLOx**	62.8%	45.9%	8.94	73.1
**Ours**	**60.4**%	53.6%	10.73	80.6

### Experimental results on the Pascal VOC dataset

#### Pascal VOC dataset

In addition to the detection of the BDD100K pedestrian dataset, this paper further validates the generalisation capability of the proposed model using the Pascal VOC dataset. The dataset includes Pascal VOC2007 and Pascal VOC2012, with a total of 20 target classes, 13690 training images and 3422 test images.

#### Experimental results and analysis

**Fig 12:**
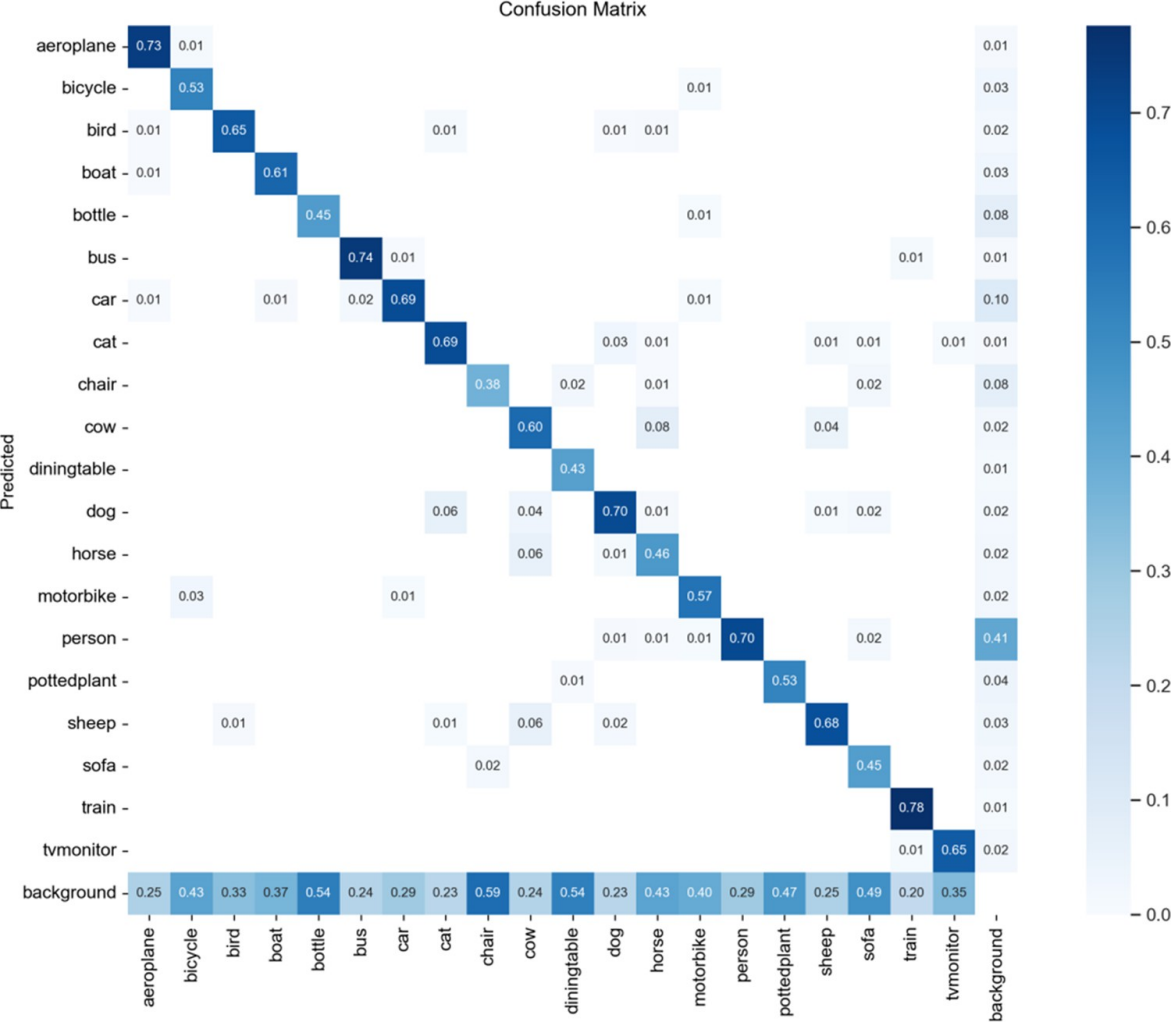
Confusion matrix diagram.

In Pascal VOC data set, this paper evaluates performance indicators through confusion matrix, and the results of confusion matrix are shown in Fig 12.

Table 3 gives the results of the single class comparison between the improved algorithm and the benchmark model on the PASCAL VOC 2007 test set. The single class AP values of the improved method are all higher than the detection values of the benchmark model, and the detection accuracy of the improved method is all better than that of the image targets detected by YOLOV5s. The improved model also has better detection capability in multi-category target detection tasks, reflecting the generality of the model, which can be adapted to different datasets or scenarios.

**Table 3:** Testing single category AP test results.

Category	YOLOV5(%)	Ours(%)
**Areoplane**	79.9	88.7
**Bicycle**	52.9	54.8
**Bird**	66.2	75.4
**Boat**	57.0	66.7
**Bottle**	45.2	52.1
**Bus**	77.6	78.9
**Car**	64.3	68.6
**Cat**	82.2	86.2
**Chair**	53.8	60.8
**Cow**	60.8	62.0
**Diningtable**	58.9	72.6
**Dog**	79.2	82.5
**Horse**	58.2	61.7
**Motorbike**	61.7	67.9
**Person**	66.0	71.6
**Sheep**	69.6	76.6
**Potteplant**	57.3	60.3
**Sofa**	53.3	60.7
**Train**	83.9	88.9
**Tvmonitor**	58.4	66.3

In order to further verify the performance of the improved model, the proposed method is compared with lightweight networks such as YOLOV3-tiny and YOLOV4-tiny, and the results are shown in Table 4. Compared with YOLOV3-tiny and YOLOV4-tiny, the accuracy of the proposed method is improved by 10.4% and 8.5%, respectively.

**Table 4:** Comparison of lightweight network performance.

Model	mAP@0.5 (%)	mAP@0.5:0.95 (%)	Params (M)	Flops (G)	FPS
**YOLOV3-tiny**	56.4	30.9	8.67	12.9	178.2
**YOLOV4-tiny**	58.3	34.7	5.88	16.2	149.5
**YOLOV5s**	65.5	44.0	7.06	16.0	153.8
**ours**	66.8	45.1	10.77	19.0	80.6

**Fig 13:**
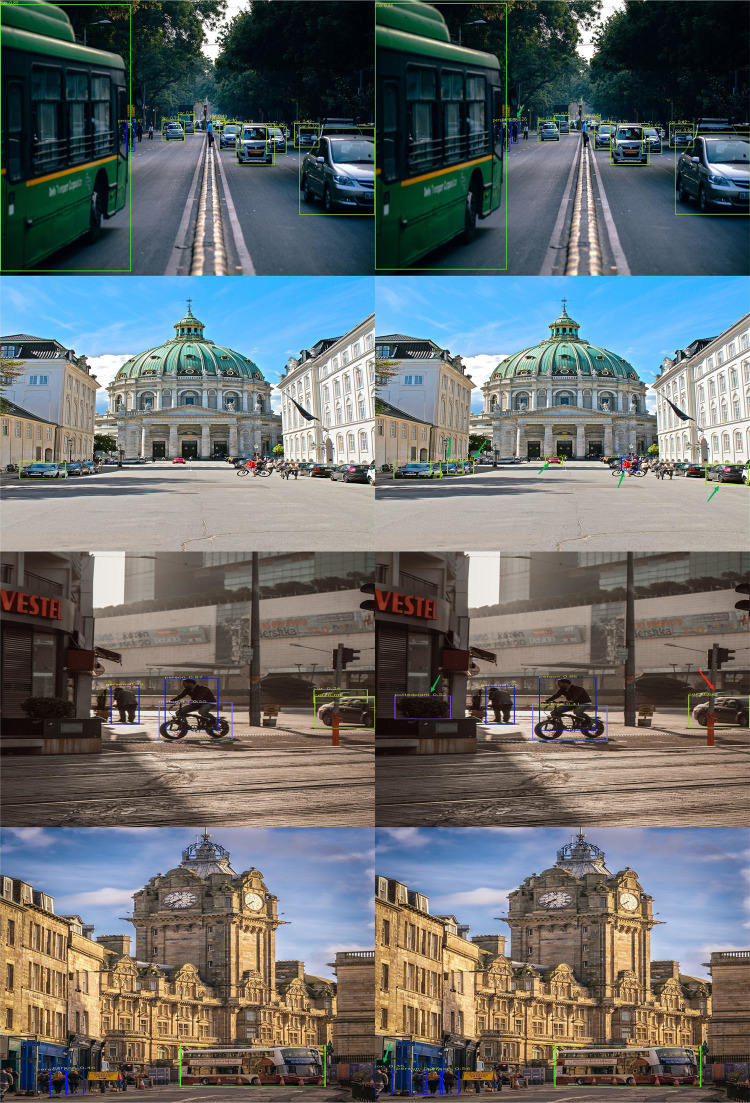
The comparison of detection results.

The results on the VOC dataset show the performance of the method compared to YOLOV5s as shown in Fig 13. The detection accuracy of the method is 66.8%, which is 1.3% higher than YOLOV5s, but the number of model parameters increases slightly due to the larger operations brought about by the correlation of using large kernel convolutions for operations. The top image is the detection result of YOLOV5s, and the bottom image is the detection result of the improved algorithm in this paper.

The performance comparison between the improved algorithm and YOLOV5s on the data set is shown in Fig 13. In common scenes and complex scenes, the proposed method is not significantly different from YOLOV5s. However, when detecting the same object, the improved model has more accurate detection effect and can better mark the location and size. It indicates that the improved algorithm has the capability of target detection compared with YOLOV5s. The blue arrow indicates that the target is not detected or the detection and positioning is not accurate, and the red arrow indicates that the predicted result of the improved algorithm is closer to the actual situation, but there are still cases of no detection. In addition to the BDD100K pedestrian data set experiment, the improved model also uses Pascal VOC data set to evaluate the proposed method. The experimental results show that the proposed algorithm can effectively detect the target objects of real pedestrians, improve the accuracy of pedestrian detection, and also has better performance on the public target detection data set, reflecting the applicability of the model detection.

## Conclusion

In this paper, an algorithm that integrates large kernel attention and the lightweight YOLOV5 model is proposed, which addresses the localization and detection requirements of pedestrian targets on the road. The proposed algorithm is based on the lightweight YOLOV5 model, incorporating attention fusion techniques. The C3LKA module is employed to capture spatial adaptability and long-distance dependencies, while the CA_NCBAM module focuses on target feature information to enhance the model’s detection accuracy. Additionally, the alpha CIOU loss function is utilized to improve the regression performance of boundary boxes and enhance positioning accuracy. To evaluate the effectiveness of the proposed algorithm based on them, experiments are conducted on two distinct datasets. The results demonstrate that the algorithm offers several advantages, including a simplified structure, low complexity, and high detection accuracy. Furthermore, the method exhibits strong generality and performs well in both pedestrian-specific and general target detection datasets. In future work, research directions will explore the integration of Transformer [29] in the latest visual field of the network. This aims to leverage the benefits of Transformer architectures and further optimize the network structure to meet the storage and computational requirements of resource-constrained device platforms. The balance between detection accuracy and detection time in target detection is often considered, so as to improve the detection effect and keep the reasoning operation time relatively stable, we can learn the model proposed by Ji et al. [30] proposed a long short-term memory recursive network model (LSTM) such as EMSN, which can help us carry out subsequent deployment and integration of pedestrian detection to improve detection efficiency. And utilizing hardware acceleration and neuromorphic computation [31,32] to help us realize the tradeoff between accuracy and time cost. Additionally, efforts will be made to collect a more extensive and complex pedestrian data set, considering the presence of vehicles on the road [33]. This will contribute to advancing the accuracy and practicality of the model.

## Supporting information

S1 Fig The architecture of the YOLOV5 mothod. (PDF)

S2 Fig Bottleneck network in C3 module. (PDF)

S3 Fig The architecture of the improved YOLOV5 mothod. (PDF)

S4 Fig Structure diagram of two LKA modules. (PDF)

S5 Fig The convolutional layer. (PDF)

S6 Fig The Ghost module. (PDF)

S7 Fig The framework of CA_NCBAM. (PDF)

S8 Fig Normalization channel attention module. (PDF)

S9 Fig The Pedestrians images. (PDF)

S10 Fig The experimental results of each model on BDD100K dataset. (a) The precision change curve (b) The curve of change in mAP@0.5.
(PDF)

S11 Fig Experimental results of different scene models. (a) Result of YOLOV5 (b) Algorithm testing results in this paper.
(PDF)

S12 Fig Confusion matrix diagram. (PDF)

S13 Fig The comparison of detection results. (PDF)

S1 Table The results of BDD100K under different models. (PDF)

S2 Table Results of different models on BDD100K dataset. (PDF)

S3 Table Testing single category AP test results. (PDF)

S4 Table Comparison of lightweight network performance. (PDF)

S1 Appendix The license of the images. (PDF)
