# Supplementary figures and images for: Pedestrian detection algorithm integrating large kernel attention and YOLOV5 lightweight model
Source: PLoS One. 2023 Nov 29;18(11):e0294865. doi: 10.1371/journal.pone.0294865 (PMC10686420; doi:10.1371/journal.pone.0294865)

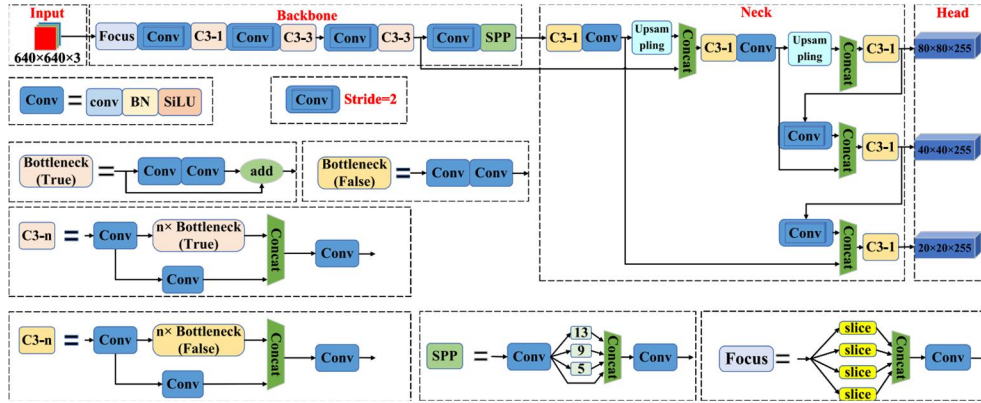

Supplement: S1 Fig — (PDF) [file pone.0294865.s001.pdf]

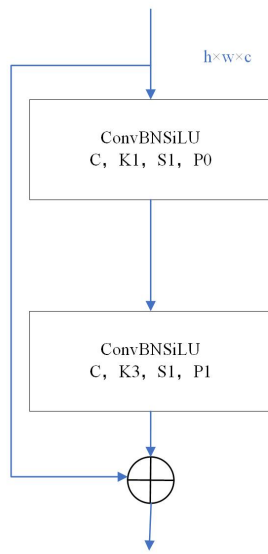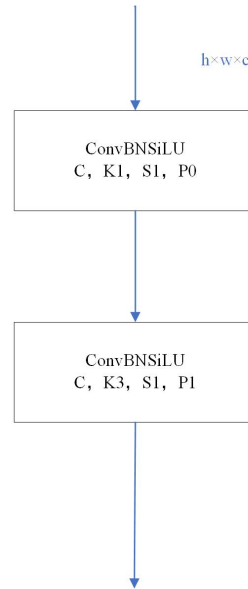

Supplement: S2 Fig — (PDF) [file pone.0294865.s002.pdf]

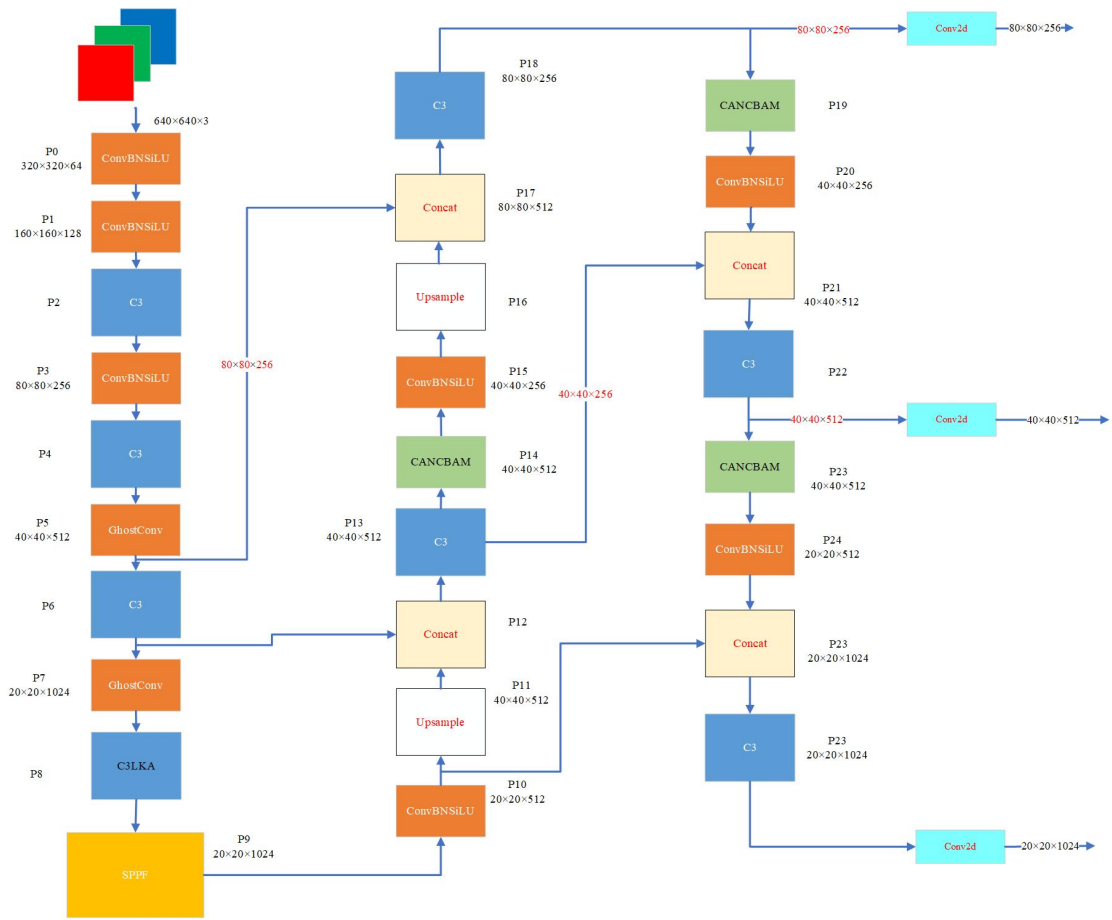

Supplement: S3 Fig — (PDF) [file pone.0294865.s003.pdf]

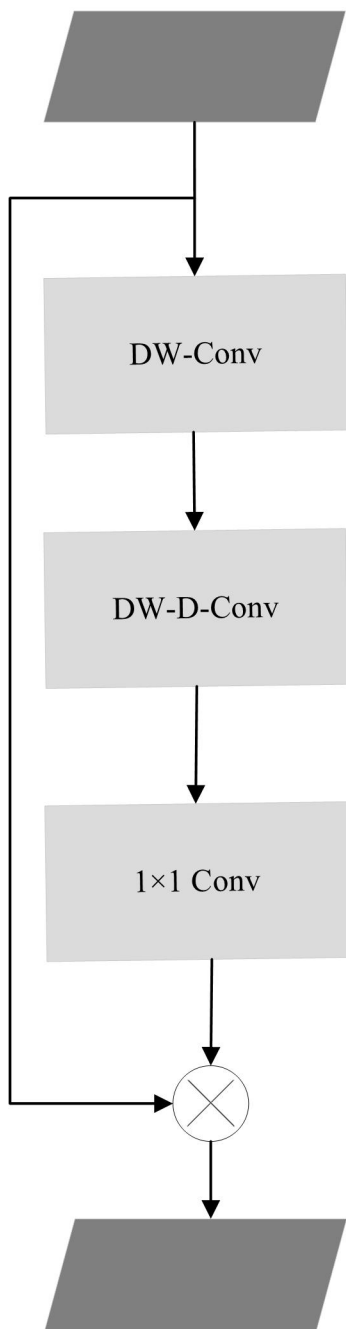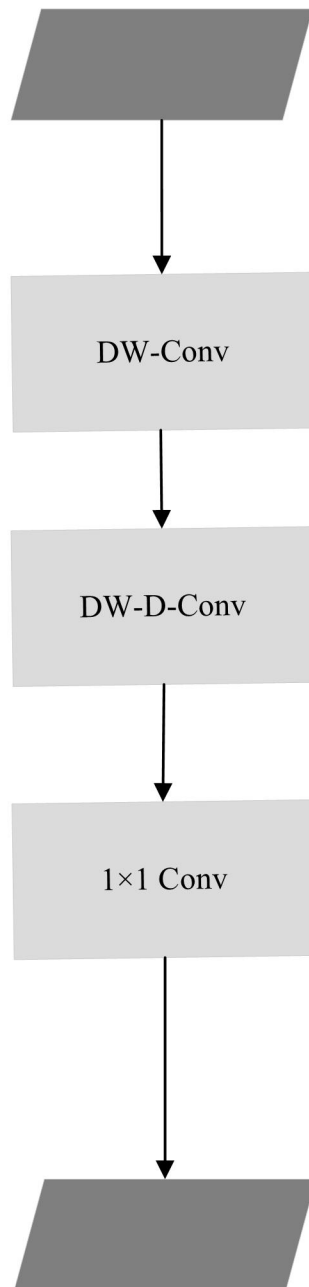

Supplement: S4 Fig — (PDF) [file pone.0294865.s004.pdf]

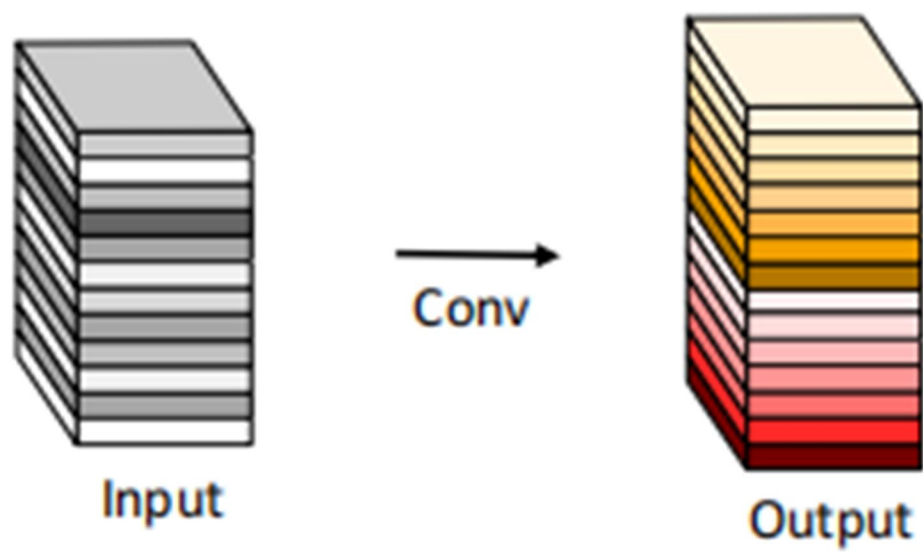

Supplement: S5 Fig — (PDF) [file pone.0294865.s005.pdf]

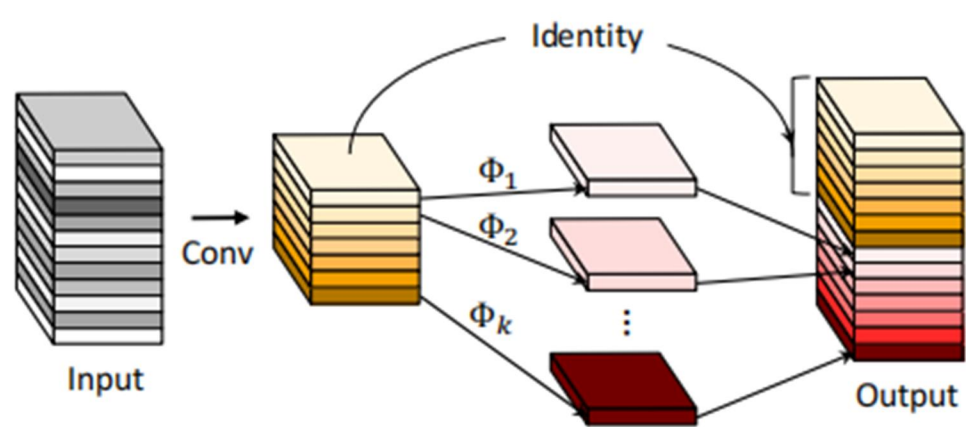

Supplement: S6 Fig — (PDF) [file pone.0294865.s006.pdf]

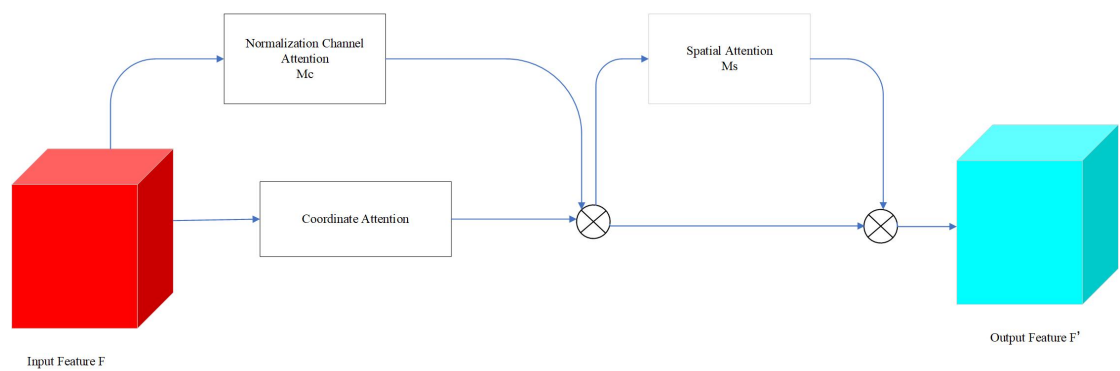

Supplement: S7 Fig — (PDF) [file pone.0294865.s007.pdf]

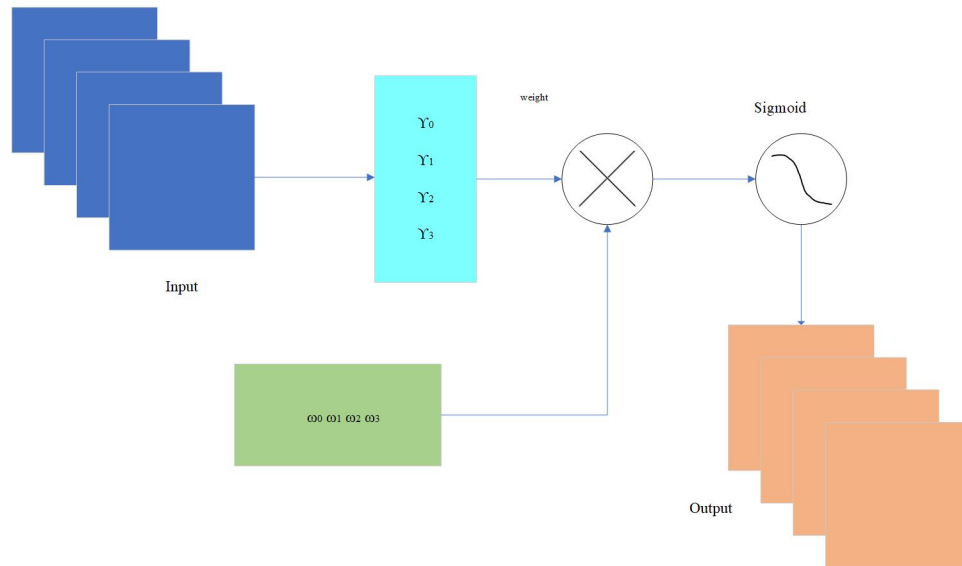

Supplement: S8 Fig — (PDF) [file pone.0294865.s008.pdf]

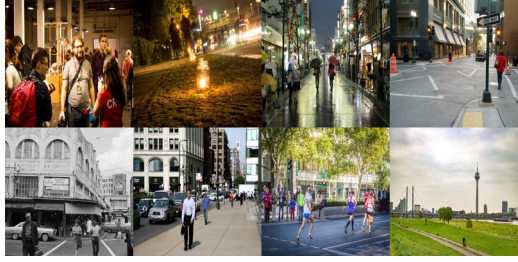

Supplement: S9 Fig — (PDF) [file pone.0294865.s009.pdf]

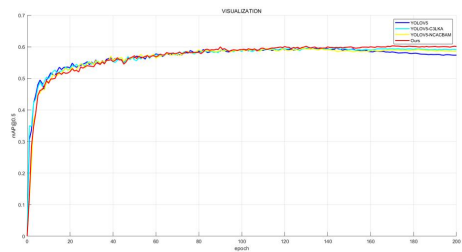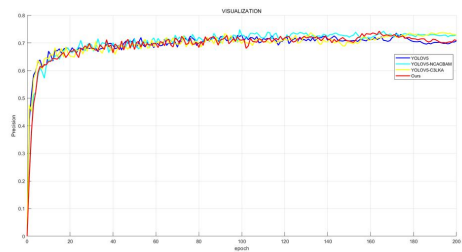

Supplement: S10 Fig — (a) The precision change curve (b) The curve of change in mAP@0.5. (PDF) [file pone.0294865.s010.pdf]

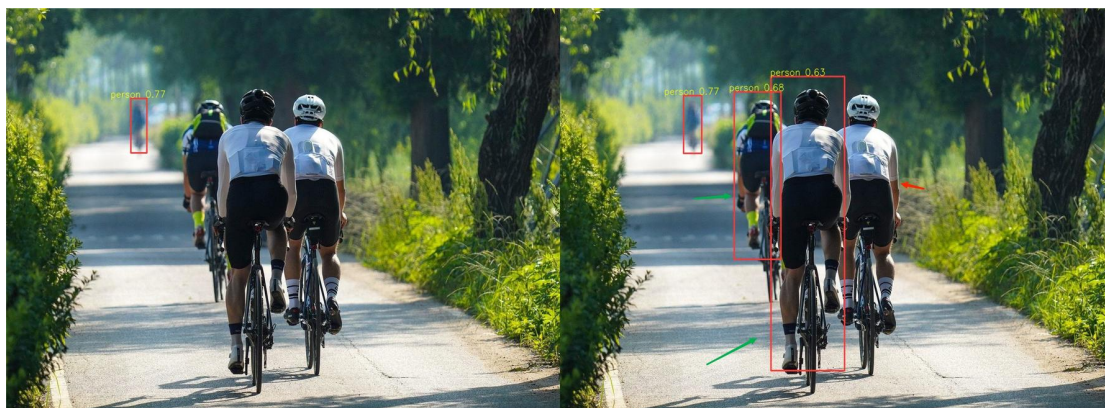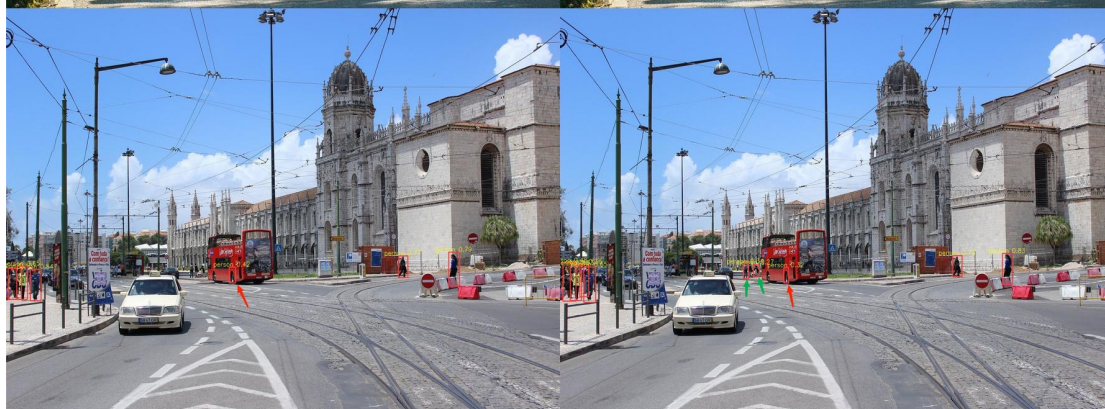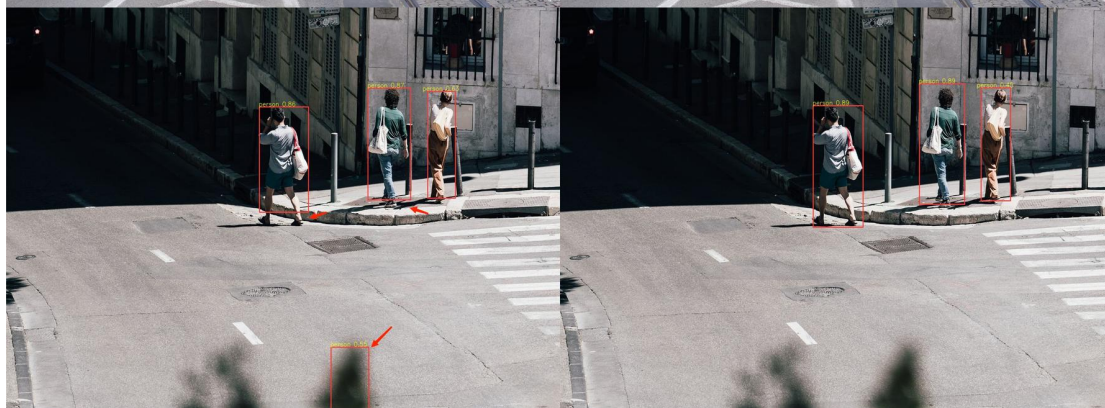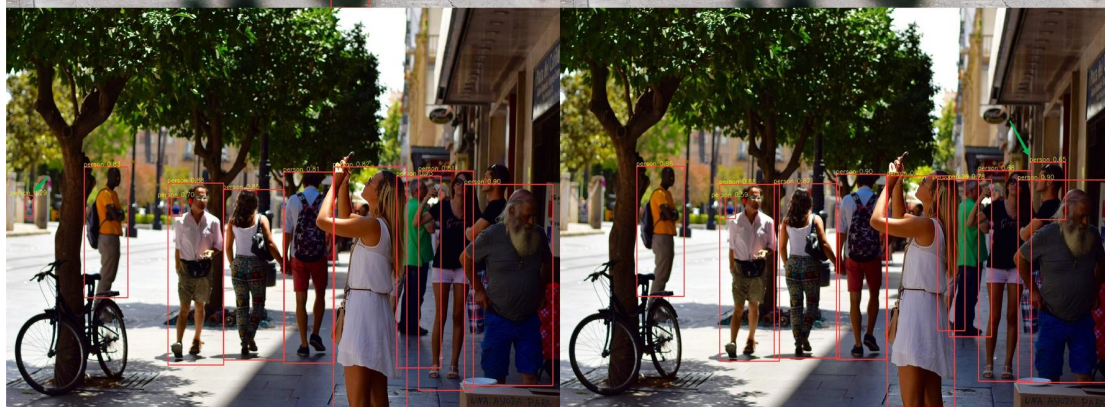

Supplement: S11 Fig — (a) Result of YOLOV5 (b) Algorithm testing results in this paper. (PDF) [file pone.0294865.s011.pdf]

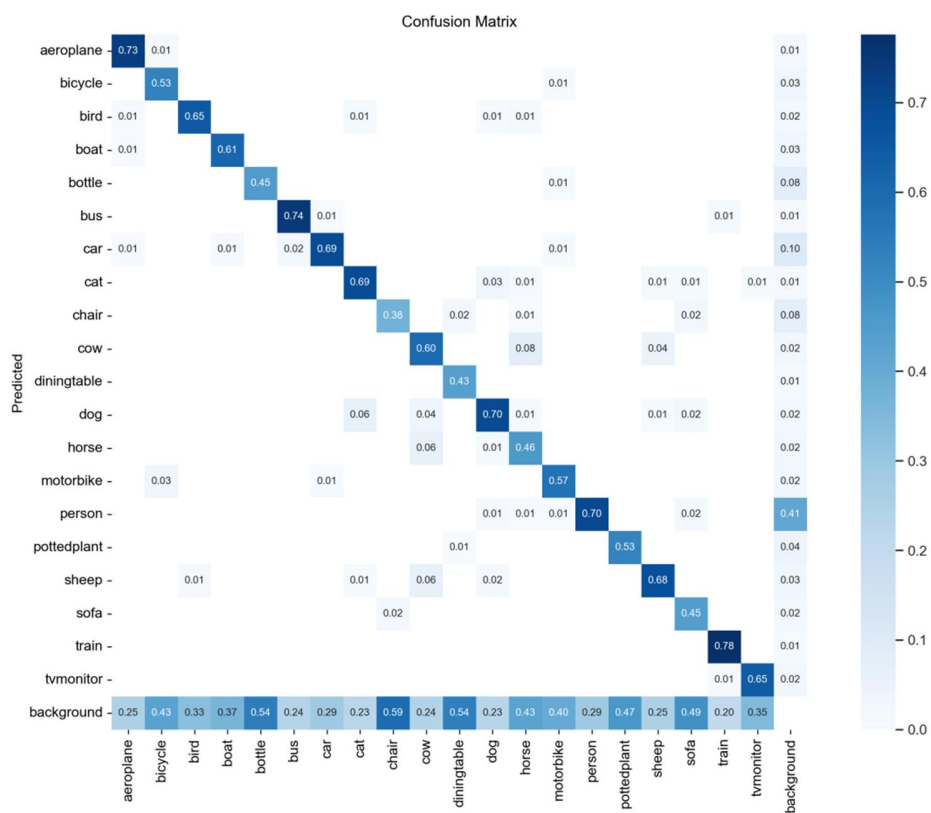

Supplement: S12 Fig — (PDF) [file pone.0294865.s012.pdf]

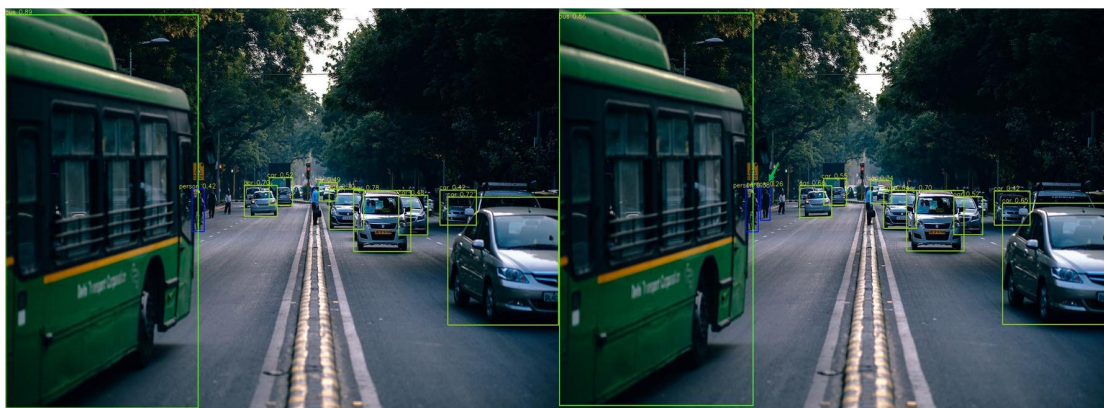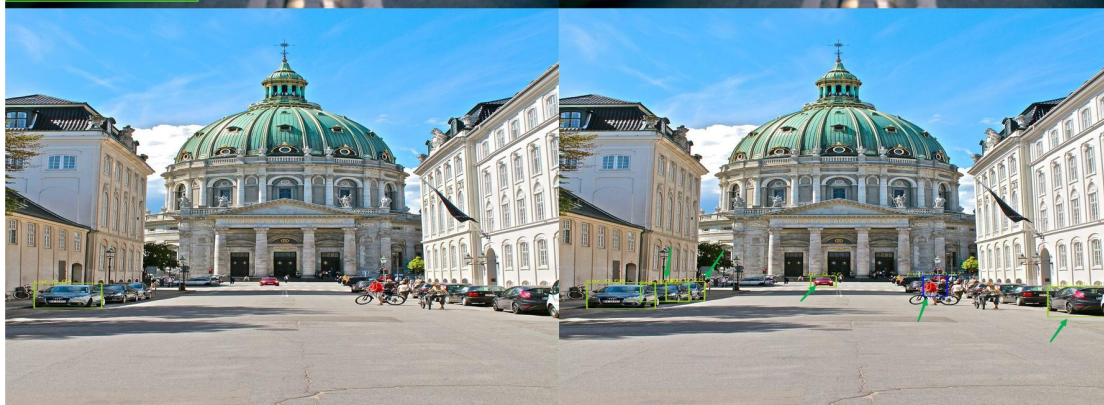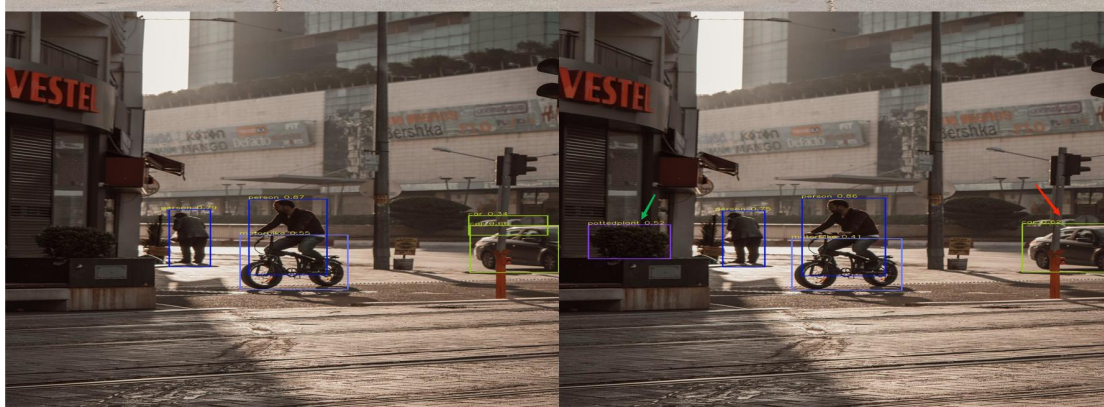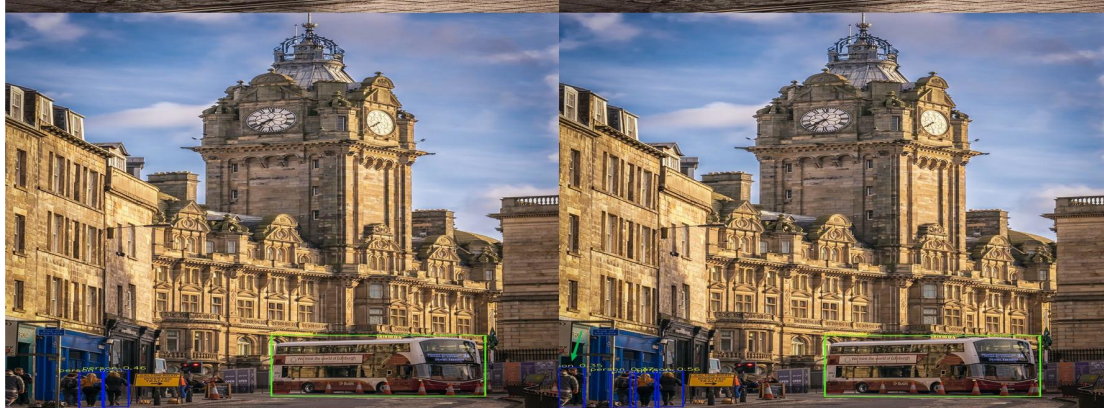

Supplement: S13 Fig — (PDF) [file pone.0294865.s013.pdf]
