# Supplementary material for: Pedestrian detection algorithm integrating large kernel attention and YOLOV5 lightweight model
Source: PLoS One. 2023 Nov 29;18(11):e0294865. doi: 10.1371/journal.pone.0294865 (PMC10686420; doi:10.1371/journal.pone.0294865)
Supplement: S1 Table — (PDF) [file pone.0294865.s014.pdf]

Model	mAP@0.5 (%)	**Precision (%)**	**Params (M)**	**Flop (G)**	**FPS**
**YOLOV5**	59.3	71.6	7.01	15.8	153.84
**YOLOV5+C3_LKA**	59.7	72.5	11.27	19.2	89.29
**YOLOV5+CA_NCBAM**	59.5	73.1	7.05	15.9	101.01
**YOLOV5+alpha CIOU**	59.5	69.9	7.01	15.8	153.8
**ours**	**60.4**	73	10.72	18.8	80.65
