# Supplementary material for: Pedestrian detection algorithm integrating large kernel attention and YOLOV5 lightweight model
Source: PLoS One. 2023 Nov 29;18(11):e0294865. doi: 10.1371/journal.pone.0294865 (PMC10686420; doi:10.1371/journal.pone.0294865)
Supplement: S2 Table — (PDF) [file pone.0294865.s015.pdf]

Model	mAP@0.5	AR	Params(M)	FPS
**Faster R-CNN**	50.04%	53.10%	3.01	/
**YOLOV7**	59.20%	53.60%	36.48	76.3
**YOLOV3-SPP**	61.60%	55.90%	62.55	49.5
**YOLOV5**	59.30%	52.60%	7.01	153.8
**YOLOx**	62.80%	45.90%	8.94	73.1
**Ours**	**60.40%**	53.60%	10.73	80.6
