# Supplementary material for: Pedestrian detection algorithm integrating large kernel attention and YOLOV5 lightweight model
Source: PLoS One. 2023 Nov 29;18(11):e0294865. doi: 10.1371/journal.pone.0294865 (PMC10686420; doi:10.1371/journal.pone.0294865)
Supplement: S3 Table — (PDF) [file pone.0294865.s016.pdf]

Category	YOLOV5(%)	Ours(%)
Areoplane	79.9	88.7
Bicycle	52.9	54.8
Bird	66.2	75.4
Boat	57	66.7
Bottle	45.2	52.1
Bus	77.6	78.9
Car	64.3	68.6
Cat	82.2	86.2
Chair	53.8	60.8
Cow	60.8	62
Diningtable	58.9	72.6
Dog	79.2	82.5
Horse	58.2	61.7
Motorbike	61.7	67.9
Person	66	71.6
Sheep	69.6	76.6
Potteplant	57.3	60.3
Sofa	53.3	60.7
Train	83.9	88.9
Tvmonitor	58.4	66.3
