# Supplementary material for: Pedestrian detection algorithm integrating large kernel attention and YOLOV5 lightweight model
Source: PLoS One. 2023 Nov 29;18(11):e0294865. doi: 10.1371/journal.pone.0294865 (PMC10686420; doi:10.1371/journal.pone.0294865)
Supplement: S4 Table — (PDF) [file pone.0294865.s017.pdf]

Model	mAP@0.5 (%)	mAP@0.5:0.95 (%)	Params (M)	Flops (G)	FPS
**YOLOV3-tiny**	56.4	30.9	8.67	12.9	178.2
**YOLOV4-tiny**	58.3	34.7	5.88	16.2	149.5
**YOLOV5s**	65.5	44	7.06	16	153.8
**ours**	66.8	45.1	10.77	19	80.6
