# Supplementary material for: Pedestrian detection algorithm integrating large kernel attention and YOLOV5 lightweight model
Source: PLoS One. 2023 Nov 29;18(11):e0294865. doi: 10.1371/journal.pone.0294865 (PMC10686420; doi:10.1371/journal.pone.0294865)
Supplement: S1 Appendix — (PDF) [file pone.0294865.s018.pdf]

Copyright about Fig9

Supporting

Here are the licenses for the eight images

1、"pedestrian paradise in the rain" by keroyama is licensed under CC BY 2.0.

2、"Chicago (ILL) Near West Side, West Jackson Blvd (Road 66) ' pedestrians '" by (vincent desjardins) is licensed under CC BY 2.0.

3、"Pedestrians at Pike Place Market, 1968" by Seattle Municipal Archives is licensed under CC BY 2.0.

4、"Striped for diagonal scramble crossing, but diagonal pedestrian signals don't work" by Eric Fischer is licensed under CC BY 2.0.

5、"42. Berlin Marathon km35" by sebaso is licensed under CC BY 4.0.

6、"20161002 IMG_5387 by sebaso" by sebaso is licensed under CC BY 4.0. To view a copy of this license, visit https://creativecommons.org/licenses/by/4.0/?ref=openverse.

7、"20170407 Düsseldorf IMG_0761 by sebaso" by sebaso is licensed under CC BY 2.0.

8、Lichterkette Berlin by @sebaso CCBY4.0
